# Omics-Derived Prognostic Biomarkers in Tongue Squamous Cell Carcinoma: A Systematic Review with Risk-of-Bias Appraisal and Translational Prioritization

**DOI:** 10.3390/cimb48040389

**Published:** 2026-04-10

**Authors:** Ioannis Astreidis, Ilias Kostidis, Andigoni Malousi, Konstantinos Paraskevopoulos, Dimitrios Andreadis, Konstantinos Vahtsevanos, Ioannis Vizirianakis

**Affiliations:** 1 Department of Oral & Maxillofacial Surgery, Specialized Cancer Treatment and Reconstruction Centre, General Hospital of Thessaloniki “George Papanikolaou”, Aristotle University of Thessaloniki, 570 10 Thessaloniki, Greece; astrimax23@gmail.com (I.A.); vaxtseva@gmail.com (K.V.); 2 Laboratory of Pharmacology, School of Pharmacy, Aristotle University of Thessaloniki, 541 24 Thessaloniki, Greece; kostiilias@pharm.auth.gr (I.K.); or vizirianakis.i@unic.cy (I.V.); 3 Laboratory of Biological Chemistry, School of Medicine, Aristotle University of Thessaloniki, 541 24 Thessaloniki, Greece; andigoni@auth.gr; 4 Department of Oral Medicine/Pathology, School of Dentistry, Aristotle University of Thessaloniki, 541 24 Thessaloniki, Greece; dandrea@dent.auth.gr; 5 Department Health Sciences, School of Life & Health Sciences, University of Nicosia, 2417 Nicosia, Cyprus

**Keywords:** tongue squamous cell carcinoma, TSCC, molecular biomarkers, prognostic biomarkers, prognosis

## Abstract

Tongue squamous cell carcinoma (TSCC) is clinically heterogeneous, and patients with a similar TNM stage can experience markedly different outcomes. We systematically reviewed omics-driven studies to identify prognostic TSCC biomarkers. Although fundamentally prognostic, we discussed their theoretical translational relevance regarding future clinical decisions—such as treatment stratification or surveillance intensity—while strictly framing them as preliminary, hypothesis-generating targets. PubMed, Scopus, Web of Science, and Cochrane were searched for original human studies published between 2014 and 2024 using high-throughput genomic or transcriptomic profiling. Study selection followed referred Reporting Items for Systematic Reviews and Meta-Analyses (PRISMA), data were extracted with a structured workbook, and risk of bias was assessed using QUIPS and PROBAST, with reporting completeness appraised using REMARK. Seventeen studies were included, identifying 85 distinct biomarkers. Across biomarkers supported by multivariable overall survival analyses, higher-risk associations were reported for *NELL2*, *PDE4D*, *CTTN*, *HBEGF*, and *CA9*, whereas lower-risk associations were reported for *AC139530.1*, *LINC01711*, *CCDC96*, *CYP2J2*, and *SPAG16*. Recurrent biological themes included IL-17 signaling, ECM-receptor interaction, and focal adhesion. *CA9* was the only biomarker reported in more than one included study, supporting its prioritization for validation. Although the evidence remains heterogeneous and largely hypothesis-generating, these markers may support the future validation of response-oriented therapeutic stratification in TSCC.

## 1. Introduction

Oral cavity cancers represent a substantial global burden within head and neck malignancies. In 2022, the IARC/WHO GLOBOCAN estimates for lip and oral cavity cancer reported 389,846 new cases and 188,438 deaths worldwide, ranking 16th for incidence and 15th for cancer mortality, with an age-standardized incidence rate (ASR) of 4.0 per 100,000 and an ASR mortality of 1.9 per 100,000. The burden is geographically uneven: Asia accounted for 66.3% of incident cases and 75.1% of deaths [1]. The oral tongue represents the most common site of oral cavity cancer, with over 90% of malignancies being squamous cell carcinomas, typically treated with primary surgery and neck dissection, followed by adjuvant therapy when indicated [2,3].

Tongue squamous cell carcinoma (TSCC) is therefore a clinically important subset of oral cavity squamous cell carcinoma (SCC). Its incidence has been reported to increase in several settings, including rising trends of oral tongue SCC among younger adults in population-based analyses [2,3]. TSCC often shows aggressive behavior and unfavorable outcomes, and patients within the same TNM stage may experience markedly different clinical trajectories [4].

Accurate prognosis is essential for treatment planning in TSCC. TNM staging remains central, yet patients with the same stage—including early-stage disease—can experience markedly different outcomes [5]. Clinicopathologic features such as tumor differentiation, perineural invasion, and lymphovascular invasion provide prognostic information but do not fully resolve risk stratification [6], supporting the need for additional indicators that better reflect tumor biology. Importantly, prognostic biomarkers are clinically meaningful only insofar as they can inform concrete management choices in TSCC, particularly (i) the management of the clinically node-negative (cN0) neck in early-stage disease (e.g., elective neck dissection versus observation), where an improved prediction of occult nodal disease and recurrence risk could reduce both undertreatment and overtreatment; (ii) the selection of post-operative adjuvant therapy intensity after definitive surgery (RT versus CRT), where risk refinement could support escalation or de-escalation beyond conventional clinicopathologic factors; and (iii) tailoring surveillance intensity, where more accurate risk estimates may rationalize follow-up schedules and imaging.

High-throughput molecular profiling has enabled the discovery of many candidate biomarkers linked to carcinogenesis and outcome [7,8]. However, TSCC-specific prognostic evidence remains fragmented, and most candidates have not been translated into routine clinical use; the current international guidelines for head and neck/oral cavity squamous cell carcinoma rely primarily on TNM staging and established pathologic risk factors to guide risk stratification and management, and they have not yet recommended molecular biomarkers for routine prognostic assessment in TSCC [6]. Many reviews have indirectly addressed prognosis through broad-spectrum studies aiming to examine tongue cancer in general, diagnostic, prognostic, and therapeutic biomarkers, or studies including other anatomical subsites, like the oral cavity and base of the tongue. Others focus mainly on one biomarker, such as E-Cadherin [9], or a class of biomarkers like microRNAs (miRNAs) [7], some of which correlate with survival outcomes. Similar conclusions were supported by additional studies [10].

A comprehensive 30-year review by Almangush et al. covering 1985–2015 [11] identified VEGF-A and cyclin D1 as the most promising immunohistochemistry (IHC)-based prognostic biomarkers in TSCC after reviewing and meta-analyzing 25 studies. However, most studies in that evidence base were IHC-driven and therefore differ fundamentally from unbiased omics workflows. In IHC discovery studies, biomarkers are typically pre-selected a priori (hypothesis-driven), and results may vary with antibody choice, staining protocols, scoring systems, and cut-off definitions, all of which can introduce measurement variability and limit cross-study comparability.

By contrast, genome-wide transcriptomic/genomic profiling enables the simultaneous, high-dimensional measurement of molecular biomarkers, supports the data-driven discovery of previously unconsidered candidates, and facilitates pathway-level and multi-gene risk modeling under a single analytic framework. Importantly, IHC remains central in routine pathology and is highly valuable for protein-level and spatial validation of omics-derived candidates.

In this systematic review, we focused on studies in which biomarker discovery was driven by high-throughput genomic or transcriptomic profiling and prognostic outcomes were reported, while allowing targeted assays (e.g., RT-qPCR and immunohistochemistry) as validation steps. We aimed to (1) catalog and classify TSCC prognostic biomarkers and multi-gene signatures identified using omics workflows, (2) summarize prognostic effect estimates with emphasis on OS and multivariable analyses, (3) appraise study quality using QUIPS/PROBAST and reporting completeness using REMARK, and (4) synthesize recurring pathway-level mechanisms that could link biomarkers to aggressive TSCC biology. In addition, we explored the theoretical implications of these markers for future research regarding treatment stratification, cN0 neck management, adjuvant therapy selection, and surveillance intensity. We strictly frame these concepts as preliminary and hypothesis-generating, based solely on their prognostic associations. Furthermore, while we discussed their theoretical potential as molecular indicators of anti-tumoral response, we explicitly acknowledge that translating these early prognostic signals into actionable clinical predictors remains an aspirational goal requiring rigorous prospective validation. Unlike prior TSCC biomarker reviews that primarily enumerate reported associations, this work integrates prognostic findings with structured risk-of-bias appraisal and a translation-oriented interpretation, moving from a flat catalog toward a prioritized shortlist of candidates and pathways most suitable for future validation in clinically and treatment-annotated cohorts.

## 2. Materials and Methods

### 2.1. Protocol and Registration

This systematic review was conducted in accordance with the Preferred Reporting Items for Systematic Reviews and Meta-Analyses (PRISMA) 2020 statement [12]. The completed PRISMA 2020 checklists for the abstract and the main manuscript are provided in Supplementary Tables S7 and S8, respectively. A PRISMA flow diagram was used to document screening and eligibility. Furthermore, The systematic review protocol was registered in PROSPERO with the registration number ID: CRD420250587741 (https://www.crd.york.ac.uk/PROSPERO/search), accessed on 15 March 2026

### 2.2. Eligibility Criteria

The research question was formulated using the PICO framework, focusing on prognostic molecular biomarkers in TSCC (Table 1). Inclusion criteria comprised original studies in humans investigating TSCC patients who underwent upfront (primary) surgical resection. It was strictly required that the analyzed molecular material was derived from treatment-naive surgical specimens. Consequently, studies including patients who had received neoadjuvant (induction) chemotherapy or radiotherapy prior to tissue collection were strictly excluded. Patients who subsequently received post-operative adjuvant therapies as clinically indicated were included. Importantly, eligible articles had to examine the tongue exclusively (i.e., not broader oral cavity cohorts). Studies published between 2014 and 2024 (search last updated 22 July 2024) were considered eligible. In addition, included studies were required to use omics-based molecular profiling to identify or evaluate prognostic biomarkers in TSCC and to report clinical prognostic outcomes, including overall survival (OS), disease-free survival (DFS), disease-specific survival (DSS), and/or associations with established clinicopathologic prognosticators. In this review, eligible studies could report either single biomarkers or multi-marker signatures (e.g., gene/lncRNA panels, risk scores, or small biomarker sets), provided they were derived from high-throughput genomic or transcriptomic profiling and linked to prognostic outcomes.

Exclusion criteria were applied to studies that did not meet the above requirements. Specifically, studies that examined other subsites of the oral cavity or oral squamous cell carcinoma (OSCC) without reporting tongue-specific results were excluded. Studies relying primarily on immunohistochemistry were also excluded. This exclusion was not based on methodological inferiority but was strictly to maintain alignment with the review’s specific scope: evaluating unbiased, high-throughput omics discovery workflows. By contrast, IHC-based studies typically evaluate candidates pre-selected a priori based on external hypotheses, which differs fundamentally from data-driven omics profiling. Studies in which outcomes were not purely prognostic and were primarily treatment-focused were excluded. Additional exclusions included non-English language, editorials, meta-analyses, preprints, reviews, retracted articles, systematic reviews, technical reports, and letters.

### 2.3. Information Sources and Search Strategy

**Figure 1:**
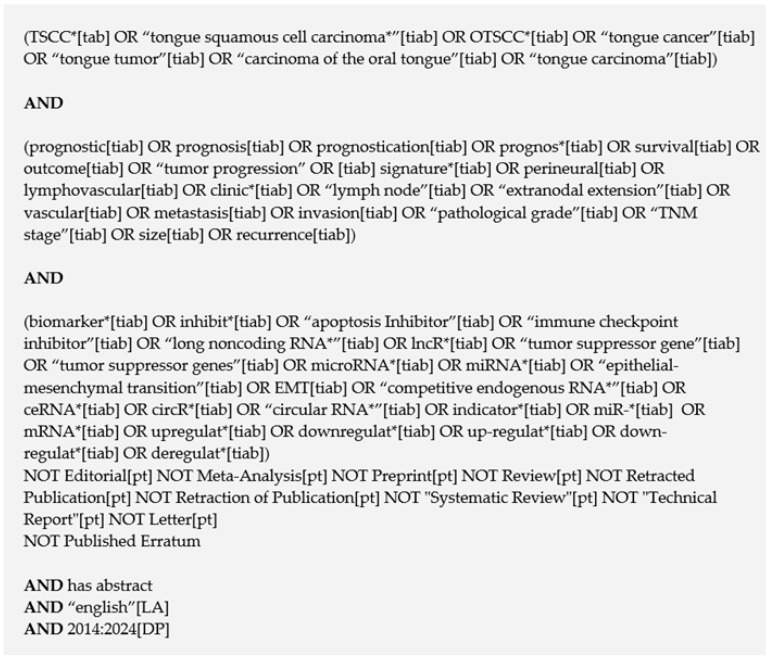
PubMed search strategy applied for the systematic review, combining terms for TSCC, prognosis, and biomarkers (final search performed on 22 July 2024). The asterisk (*) represents a truncation (wildcard) symbol used in the database search strategy to retrieve all possible word endings for a given root (e.g., ‘prognos’ retrieves prognosis, prognostic, prognosticator, etc.).

A comprehensive search strategy was implemented across four databases: PubMed, Scopus, Web of Science, and Cochrane. The final searches were conducted on 22 July 2024. Database-specific adaptations were applied to ensure compatibility with each platform’s search syntax. The search strategy combined MeSH-controlled vocabulary and free-text keywords covering the concepts of TSCC, prognosis, and biomarkers. Τhe full PubMed strategy is presented in Figure 1, and database-specific adaptations for Scopus, Web of Science, and Cochrane are provided in the Supplementary Materials (Figures S1–S3). Limits were applied to include only studies published between 2014 and 2024. This publication window was selected because, prior to 2014, study cohorts were not restricted to tongue cancer, and studies employing whole-transcriptome approaches (rather than targeted molecular panels) were not identified. Language restrictions were imposed, and filters were used to exclude non-primary research articles (e.g., reviews, editorials, letters, and conference abstracts). Gray literature was not searched or included.

As this study is a systematic review rather than an individual participant data (IPD) meta-analysis, raw gene expression datasets were not downloaded or reprocessed; instead, reported biomarkers, analytical methods, and prognostic effect estimates were extracted.

### 2.4. Study Selection and Screening Process

Two independent reviewers (IA and IK) screened all retrieved studies using the Rayyan web application [13]. Title and abstract screening were performed based on predefined eligibility criteria. Discrepancies were resolved through consensus and discussion with a third reviewer (AM). Automation tools of Rayyan were used only for deduplication; no machine learning pre-screening was applied. Reasons for exclusion at full text were recorded and are shown in the PRISMA flow diagram.

### 2.5. Data Charting Process and Data Items

Data charting was performed by IA and IK. To support consistent full-text appraisal, a shared Excel workbook was developed with a structured checklist (Table S4). Data were extracted in line with the eligibility criteria and included: author(s), sample size, biomarker(s), analytical method, pathway and pathway enrichment analyses (when reported), the prognostic association in relation to biomarker expression, and the approach used to validate the biomarker and link it to prognosis, including the specific clinicopathologic covariates adjusted for in multivariable survival models, where applicable. No missing or unclear information was identified in the core checklist fields (authors, sample size, biomarkers, analytical method, prognostic association, and method of prognostic assessment). Pathway and gene set enrichment analyses were treated as supplementary but informative variables and were therefore recorded when available and omitted only when not reported in the original study.

### 2.6. Risk of Bias and Reporting Quality Assessment

Risk of bias was assessed independently by IA and IK, with disagreements resolved by consensus and, when required, by a third reviewer (AM). Studies evaluating individual biomarkers as prognostic factors were appraised using the Quality In Prognosis Studies (QUIPS) [14] tool across six domains (study participation, study attrition, prognostic factor measurement, outcome measurement, confounding, and statistical analysis/reporting). Studies developing prognostic models (including multi-gene/lncRNA signatures, risk scores, and nomograms) were assessed using the Prediction model Risk Of Bias assessment tool (PROBAST) [15] across four domains (participants, predictors, outcome, and analysis). Domain-level judgments were recorded and summarized as an overall study judgement. Two tools were used because studies whose core translational product was a multivariable risk score, gene signature, or clinical nomogram were evaluated using PROBAST to appropriately capture risks related to model development, overfitting, and validation. Conversely, studies primarily focused on evaluating the independent prognostic value of standalone factors were assessed using QUIPS. In addition, reporting completeness was evaluated using the Reporting Recommendations for Tumor Marker Prognostic Studies (REMARK) checklist [16]; REMARK was used to assess reporting quality rather than risk of bias.

### 2.7. Data Synthesis and Analysis

Studies were grouped according to cohort design (comparisons between cancer specimens with different clinical outcomes versus tumor–normal comparisons) and biomarker class (coding versus non-coding). Because most biomarkers were evaluated in single studies and were identified using heterogeneous assays, cutoffs, and modeling strategies, quantitative pooling (meta-analysis) was not appropriate. Findings were therefore synthesized narratively and in structured tables, summarizing each biomarker’s reported prognostic association, the validation strategy used in the primary study, and molecular pathways highlighted by pathway or enrichment analyses.

Exploratory quantitative synthesis (*CA9*): When ≥2 eligible studies reported comparable multivariable hazard ratios (HRs) for the same biomarker and the same endpoint (overall survival, OS), we conducted an exploratory inverse-variance meta-analysis on log (HR). Standard errors were derived from reported 95% confidence intervals. We estimated pooled effects using both fixed-effect and random-effects (DerSimonian–Laird) models and quantified between-study heterogeneity using Cochran’s Q, I2, and tau2. Within this review, these criteria were met only for *CA9*, which was therefore the only biomarker eligible for exploratory quantitative pooling.

Where available, effect estimates for OS were extracted as hazard ratios (HRs) with 95% confidence intervals (CIs), together with the reported endpoint (OS/DFS/Recurrence-Free Survival) and the statistical approach (Kaplan–Meier/log-rank; Cox regression; univariable or multivariable). Given that OS was the most consistently reported outcome across included studies, OS was used as the primary endpoint for cross-study comparison; DFS and Recurrence-Free Survival (RFS) were recorded when available but were not used for cross-study ranking due to limited reporting. For interpretability, the comparative synthesis prioritized biomarkers supported by Cox regression, with multivariable results treated as the highest level of evidence within individual studies because they account for confounding by clinicopathologic factors. Univariable-only results were retained in the extraction tables but were not used to define the shortlist of biomarkers supported by adjusted analyses. A study characteristics table, a biomarker-level results table, and a forest plot were produced using the extracted hazard ratios and CIs. Studies that did not report sufficient information to extract at least one effect estimate (HR and CI) were included in the narrative synthesis but could not contribute to the structured effect-size summary.

For pathway synthesis, reported pathways from enrichment analyses were extracted as presented in the primary studies. To emphasize recurring biological themes, pathways supported by more than one biomarker and/or identified across multiple studies were prioritized in the main pathway table, while single-study pathways were treated as supplementary context.

### 2.8. Evidence Tier Framework and Translational Readiness Mapping

To address the need for clinical interpretability beyond a descriptive list, we applied a prespecified evidence tier framework to prioritize single biomarkers and multi-gene signatures for translational follow-up. The unit of assessment was each biomarker or multi-marker signature as reported in the included studies. Tier assignment was based on three pillars: (i) replication across independent cohorts/studies, (ii) robustness of prognostic association for overall survival (OS) under multivariable modeling, and (iii) feasibility of measurement using clinically scalable assays.

For the definition of evidence tiers, biomarkers/signatures were classified into four tiers: Tier 1 (highest readiness): reported in ≥2 independent eligible studies and supported by multivariable Cox regression for OS or equivalent adjusted survival modeling and assay-feasible using routine or near-routine platforms (e.g., IHC on FFPE, RT-qPCR, or targeted DNA testing). Tier 2: supported by multivariable OS analysis and supported by an independent validation cohort (external dataset and/or separate clinical cohort distinct from the discovery cohort) but not replicated across ≥2 eligible studies within the review. Tier 3: supported only by univariable survival modeling and/or internal-only validation (e.g., TCGA splits, resampling, cross-validation) with elevated risk of optimism/overfitting and no independent cohort confirmation. Tier 4: findings without extractable effect estimates, with unclear cut-offs, unclear confounding control, or otherwise insufficient information to support robust prognostic inference.

For operational ease, it should be defined that independent replication required that the biomarker is reported as prognostic in separate eligible publications using non-overlapping cohorts; repeated analyses of overlapping public datasets, like TCGA re-analyses, were not considered independent replication. Independent validation cohort refers to validation in a cohort distinct from the discovery dataset, such as a separate GEO series, a separate institutional cohort, or a clearly defined external dataset. Assay feasibility was classified as feasible if a marker could plausibly be implemented with standard pathology/molecular workflows (FFPE-based IHC, RT-qPCR, or targeted DNA testing). Signatures requiring whole-transcriptome sequencing/microarray and bespoke computational pipelines were considered lower feasibility unless a clinically deployable panel strategy was explicitly supported.

For each prioritized biomarker/signature, we mapped the reported prognostic signal to one or more TSCC clinical decision points (cN0 neck management, adjuvant therapy intensity, surveillance frequency). This mapping was not intended to imply clinical actionability from the current evidence; rather, it operationalized a context-of-use hypothesis indicating where a prognostic signal could be most relevant if subsequently validated.

Mapping decisions were pre-specified using the following criteria: (i) A biomarker was mapped to neck management when the primary evidence suggested an association with nodal or occult nodal metastasis, regional recurrence, or invasion–metastasis propensity, or when the discovery design explicitly contrasted N0 vs. N+ cohorts. Biomarkers/signatures linked to metastatic biology (e.g., ECM–receptor interaction, focal adhesion, invasion programs) were mapped to this decision point because they plausibly relate to the risk of regional spread that drives elective neck treatment. (ii) A biomarker was mapped to adjuvant decision-making when the evidence supported association with overall survival (OS) or disease-specific survival (DSS) in multivariable models, and/or when the implicated biology plausibly reflects radio- or chemoresistance or aggressive systemic risk. (iii) A biomarker was mapped to surveillance when the reported signal related to recurrence risk (DFS/RFS), early progression, or aggressive biology likely to manifest as early failure, and when the biomarker could plausibly stratify patients into higher versus lower near-term risk groups (i.e., those who might benefit from closer follow-up schedules).

Evidence tiers were interpreted alongside QUIPS/PROBAST domain judgements. In particular, a high risk of bias in confounding control (QUIPS) or model analysis/overfitting (PROBAST) was recorded as a key limitation in translational readiness, and such limitations were explicitly flagged in the translational readiness table. In addition, each prioritized biomarker/signature was mapped to a plausible TSCC clinical decision point: (i) management of the clinically node-negative (cN0) neck in early-stage disease (elective neck dissection versus observation), (ii) post-operative adjuvant therapy intensity (RT versus CRT), and/or (iii) surveillance intensity. Evidence tiers and translational readiness fields were assigned independently by two reviewers (IA and IK), with disagreements resolved by consensus and consultation with a third reviewer (AM) when required.

## 3. Results

### 3.1. Study Selection

**Figure 2:**
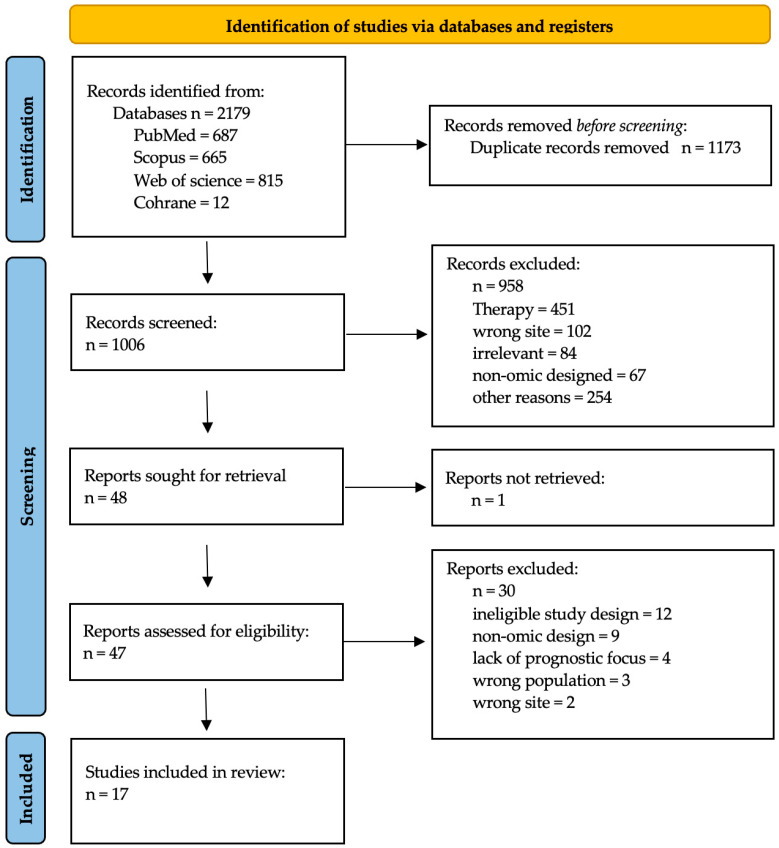
PRISMA 2020 flow diagram.

From the database search, in total, 2179 records were retrieved, including 687 from PubMed, 665 from Scopus, 815 from Web of Science, and 12 from Cochrane. All records retrieved from the four databases were imported into Rayyan (rayyan.ai) [13] for deduplication, leaving 1006 unique records. The title and abstract screening excluded 958 records (including non-omic studies, irrelevant sites, and therapy-focused articles), leaving 48 reports sought for full-text retrieval. One report could not be retrieved, resulting in 47 reports assessed for full-text eligibility. Of these, 30 reports were excluded due to ineligible study design, yielding a final set of 17 included studies. The complete list of the 30 excluded full-text reports, along with the specific reasons for their exclusion, is provided in Supplementary Table S6. The screening workflow is illustrated in the PRISMA flow diagram (Figure 2).

### 3.2. Characteristics of Eligible Studies

**Figure 3:**
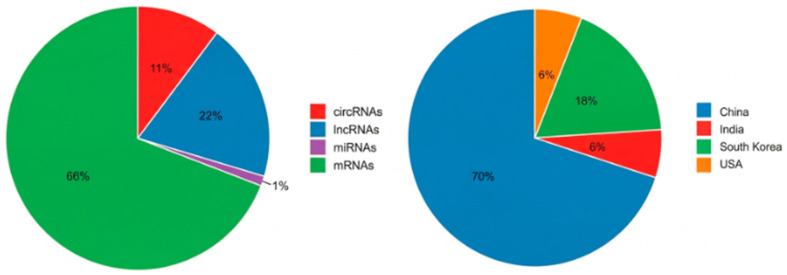
Pie charts of the molecular biomarker types and study origin.

Details of the 17 eligible studies are summarized in Table 2. Fifteen of the studies (88%) originated from East Asia, one (6%) was from North America, and one (6%) was from India (Figure 3). Most were published within the past five years (94%) and analyzed large cohorts (>100 cases), often leveraging The Cancer Genome Atlas Project (TCGA) or Gene Expression Omnibus (GEO) data. Seven studies (41%) examined correlations within cancer samples across early and late-stage diseases. Survival analysis using Kaplan–Meier curves was employed in 94% of the studies. Despite this high percentage, only 12% (two studies) incorporated a nomogram based on multivariate analysis to support clinical implementation.

### 3.3. Biomarker Types and Association with TSCC Prognosis

In total, our synthesis identified 85 distinct molecular biomarkers. The breakdown comprises 56 mRNAs (66%), 19 lncRNAs (22%), 9 circRNAs (11%), and 1 miRNA (1%) (Figure 3). The studies were grouped into two broad categories: those comparing different subgroups of TSCC tumors (cancerous vs. cancerous), mainly with patients stratified by nodal metastasis, including occult metastasis vs. patients without nodal involvement, and those comparing tumor tissue with adjacent normal mucosa (cancerous vs. normal) (Figure 3, Table S4).

Biomarker synthesis is organized biologically, reflecting the primary analytical comparisons utilized in the included studies. Specifically, Section 3.3.1 discusses prognostic markers derived from comparisons between TSCC subgroups with different clinicopathologic phenotypes, while Section 3.3.2 focuses on markers identified through tumor-to-normal tissue comparisons. To prevent the blurring of evidentiary strength within this biological narrative, all discussed biomarkers have been formally stratified into four distinct tiers of confidence which are comprehensively detailed in Section 3.6 and in Supplementary Table S5. Throughout the following sections, explicit textual signposting is utilized to distinguish single-study exploratory findings from higher-confidence, internally validated, or replicated data (Tiers 1–4).

#### 3.3.1. Comparisons Between TSCC Subgroups with Different Clinicopathologic Phenotypes

Seven studies conducted phenotype-based comparisons, most commonly contrasting node-negative (N0) versus node-positive (N1–N3; “N+”) TSCC to identify prognostically relevant molecular differences. Representing an exploratory, lower-confidence single-study association (Tier 4), Yang et al. [17] applied next-generation sequencing (NGS) with Kyoto Encyclopedia of Genes and Genomes (KEGG) pathway analysis in 19 T1–T2N+ and 22 T1–T2N0 cases and identified *TNFRSF10C* as the main differentially expressed gene (DEG) within the leukocyte transendothelial migration pathway. They subsequently validated *TNFRSF10C* copy number variants (CNVs) in the same 41-patient cohort and evaluated *TNFRSF10C* expression in 411 Cancer Genome Atlas Head–Neck Squamous Cell Carcinoma (TCGA-HNSC) cases with lymph node metastasis (LNM) and OS data; increased TNFRSF10C CNVs were associated with better DFS in N0 patients. Demonstrating a slightly higher level of single-study evidence through external in silico validation (Tier 3), Xiao et al. [18] reported that *IER3* expression was higher in patients with LNM and was associated with poorer OS and DFS; these findings were derived from microarray data through Gene Set Variation Analysis (GSVA) comparing primary tumors and metastatic cervical lymph nodes in the public cohort GSE2280 (GPL96) and were validated in 148 TSCC cases from TCGA as well as in the Tca-8113 cell line.

Yielding exploratory evidence (Tier 4) due to a lack of statistically significant prognostic validation, Lee et al. [19] used NGS with Gene Ontology (GO) and KEGG enrichment in 23 T1–T2N0 and 12 T1–T2N+ patients and found that the reduced expression of *DEFB4A*, *DEFB103B*, and *DEFB4B* was associated with nodal involvement and poorer prognosis; validation in TCGA TSCC cases (39 N0 and 31 N+) showed similar expression patterns but without statistical significance, and survival analyses were not performed. Moving to findings with moderate evidentiary support through additional cohort validation (Tier 3), in a separate study using RNA-seq-based GO/KEGG analyses, Lee et al. [20] compared 65 T4N0 and 41 T1/2N2/3 cases and reported that *ACTA1* overexpression was associated with occult metastasis; the results were supported in an additional cohort (25 T4N0 and 36 T1–2N2–3). Similarly categorized as a higher-confidence finding (Tier 2) due to multivariable validation, using a similar microarray enrichment approach, Yang et al. [21] analyzed 6 N0, 6 N+, and 12 normal controls and reported that increased *MFAP5* and *TNNC1* expression was associated with poorer DFS and higher recurrence risk; MFAP5 was additionally reported as an independent biomarker for occult LNM based on multivariable Cox regression, with validation in a cohort of 93 TSCC patients assessing DFS and recurrence.

Two studies in this subgroup focused on non-coding or genetic biomarkers. Representing a preliminary, single-study observation (Tier 4) in a cohort of 41 N0 and 35 N+ cases, Li et al. [22] reported that higher *ADAMTS9-AS2* lncRNA levels were associated with larger tumor size, advanced stage, LNM, and poorer prognosis, and they investigated a proposed ceRNA mechanism involving the miR-600/EZH2 axis and epithelial–mesenchymal transition (EMT) in functional experiments. Conversely, demonstrating stronger evidentiary support through validation in an expanded clinical cohort (Tier 3), Kim et al. [23] evaluated *TERT* promoter (*TERTp*) mutations (C228T and C250T) in 16 TSCC patients younger than 45 years and 28 patients older than 45 years, reporting associations with advanced TNM stage and reduced OS, particularly among younger patients; clinical validation was performed in an expanded cohort (96 patients <45 and 202 patients ≥45). No pathway analyses were reported in these two studies.

#### 3.3.2. Comparison of Cancerous with Normal Tissues

Ten studies compared tumor and normal tissues and reported coding and/or non-coding biomarkers associated with TSCC prognosis.

In the first one using TCGA RNA-seq data from tongue squamous cell carcinoma in 147 tumor samples and 15 normal samples, demonstrating moderate evidentiary strength through the development of a multivariable risk model and a clinical nomogram (Tier 2), Ren et al. derived their model entirely from this public cohort [24]. They first linked lncRNAs to autophagy by correlating lncRNA expression with 232 autophagy genes, then used univariate Cox to screen survival-associated lncRNAs and multivariate Cox (stepwise, minimum AIC) to select a 10-lncRNA “molecular signature” and compute a patient risk score. For prognosis, they split patients into high- vs. low-risk by the median risk score and showed significantly worse overall survival in the high-risk group, concluding that this autophagy-related lncRNA signature can predict TSCC prognosis and may represent a potential prognostic biomarker.

Elevating the level of evidence through robust statistical validation and clinical modeling (Tier 2) in another study of 127 TCGA TSCC patients and 13 normal controls [25], Liu et al. developed a 15-gene signature—including *ADTRP*, *SPAG16*, and *HBEGF*—that independently predicted shorter OS, with internal validation using the concordance index (C-index), calibration plots, and time-dependent ROC curves. They further integrated this signature into a nomogram incorporating established clinicopathologic prognosticators, demonstrating a strong potential for implementation as a prognostic tool. Returning to single-marker evaluations but supported by independent cohort validation (Tier 3), Liu et al. [26], following a microarray analysis of five TSCC and five normal samples, reported that elevated *circ_0000919* expression in tumor tissues was significantly associated with reduced OS; circ_0000919 levels correlated positively with T stage, N stage, and overall TNM classification, and these findings were validated in an additional cohort of 60 tumors and 60 normal tissues.

Two studies using tumor–normal comparisons reported single-gene or multi-gene prognostic associations. Demonstrating moderate evidentiary strength through advanced multi-dataset bioinformatic modeling (Tier 3), the first one [27] analyzed GEO data series GSE31056 and GSE34105 (23 TSCC/73 normal and 62 TSCC/16 normal tissues, respectively), used network and feature-selection approaches (WGCNA, LASSO, and an additional machine learning method as reported by the authors), and identified *SEMA3C* as a prominent gene. They applied WGCNA, a network-based approach, to identify co-expressed gene modules and LASSO regression, a method that selects a smaller set of genes to build a prognostic model; survival analyses in these datasets supported an association between higher SEMA3C expression and worse survival. Following a higher approach that yields Tier 2 evidentiary support, the other one [28] integrated public and private datasets (60 TSCC tissues and 60 matched adjacent normal tissues), supplemented with four GEO datasets (GSE13601, GSE31056, GSE9844, GSE30784; 217 tumor and 93 normal samples), and used 143 TCGA tongue cancer cases for survival analysis. By combining DEGs with ferroptosis-related genes, *CA9* (carbonic anhydrase IX), *TNFAIP3*, and *NRAS* were highlighted as candidate prognostic markers. Importantly, we noted a fundamental reporting discrepancy in the original study by Zhu et al.: while their univariate analysis (Table 5 of their paper [28]) reported hazard ratios < 1 for these markers (e.g., *TNFAIP3* HR = 0.43, *NRAS* HR = 0.47), both their narrative text and Kaplan–Meier curves paradoxically claimed an association with poorer survival (suggesting an unstated inversion of the reference group). Furthermore, in their rigorous multivariable model, only *CA9* maintained independent prognostic significance (HR = 1.263, *p* = 0.001), while *TNFAIP3* and *NRAS* failed to reach statistical significance.

Demonstrating a higher evidentiary strength through multivariable prognostic validation (Tier 2), Hu et al. [29] used TCGA transcriptomic data (145 TSCC, 15 normal tissues) with Pearson correlation to identify immune-related lncRNAs and constructed a six-lncRNA risk model (*MIR4713HG*, *AC104088.1*, *LINC00534*, *NAALADL2-AS2*, *AC083967.1*, *FNDC1-IT1*). Patients stratified into high- versus low-risk groups showed significantly different OS; the findings of this study were evaluated in a testing cohort of 52 TCGA TSCC patients, and the risk score was reported as an independent prognostic factor in uni- and multivariable Cox models. In contrast, representing a lower-confidence, exploratory finding based primarily on univariate survival associations (Tier 4), Li et al. [30] analyzed TCGA TSCC (126 tumors, 13 normal controls) to identify differentially expressed mRNAs/lncRNAs/miRNAs and linked candidates to OS using Kaplan–Meier/log-rank testing after median expression stratification; they constructed a ceRNA network (starBase and Cytoscape) to prioritize prognostic RNAs within the network and reported that lower *NAGS* (mRNA), *hsa-miR-1229-3p* (miRNA), and *AL359851.1* (lncRNA) were associated with improved outcomes. They also used ConsensusClusterPlus (top 2000 most variable genes) to define two expression-based subtypes, compared clinicopathologic features between subtypes using χ^2^ tests, and reported superior OS for Subtype B; the most upregulated genes were *BPIFB2*, *CTCFL*, and *NTS* (Subtype A) and *DEFB4B, CRNN*, and *MUC21* (Subtype B).

Demonstrating moderate evidentiary strength through the prospective clinical validation of individual markers (Tier 3), in an Indian RNA-seq study of 12 tumors and two normal tissues [31], DEGs were examined using gene set enrichment and protein–protein interaction analyses, followed by prospective validation and survival analysis in 100 TSCC tissues; elevated *TNC* and *PDPN* were associated with occult node positivity, while the expression of *MMP9*, *LAMC2*, *DSG2*, *PLAU*, *FOXM1*, and *MYO1B* were associated with treatment failure in early-stage patients. Elevating the level of evidence through immune-based risk modeling and multivariable prognostic validation (Tier 2), Jin et al. [32] analyzed TCGA TSCC RNA-seq data (147 tumors, 15 normal tissues) using ssGSEA to define three immune infiltration clusters and reported a significant OS difference across clusters; pathway and survival analyses further supported a prognostic risk model including *PGK1*, *GPI*, and *RPE*, which was additionally reported as an independent prognostic factor in multivariable Cox regression.

Finally, across eligible studies, *CA9* was the only biomarker identified in more than one study, thereby elevating it to the highest level of evidentiary confidence in our review (Tier 1) as an independently replicated prognostic signal. Wang et al. [33] analyzed TCGA TSCC (125 tumors, 11 normal) and GSE31056 (23 TSCC, 73 normal) using RNA-seq/microarray differential expression, functional enrichment, and PPI network analyses, and reported that a high CA9 expression was associated with worse prognosis in survival analysis; validation was performed in 20 TSCC specimens using RT-PCR, Western blotting, and immunohistochemistry.

Furthermore, among the seven studies that reported multivariable survival analyses, the selection of adjusted covariates varied substantially, highlighting a lack of standardized prognostic modeling. The most frequently included clinical confounders were age, gender/sex, overall clinical stage, T/N classifications, and tumor grade (e.g., [24,25,31]). However, adjustment models were highly heterogeneous. Several studies incorporated calculated multi-gene risk scores alongside standard clinical data [24,29,31]. Others utilized highly specific clinical parameters, such as neurovascular invasion [21] or smoking history [32]. Notably, certain models primarily evaluated the independent prognostic value of multiple biomarkers against each other without explicitly adjusting for baseline clinical confounders (e.g., Zhu et al. [28]).

All eligible studies are listed in Table 2. Studies are listed in the order they are discussed in the text, rather than by sequential numbering, to improve traceability between the narrative synthesis and the table.

### 3.4. Risk of Bias and Reporting Quality of Included Studies

Risk of bias assessments are summarized in Table S1 QUIPS [14] and Table S2 PROBAST [15]. Overall, 13/17 studies were assessed as having a high risk of bias, while 4/17 had a moderate risk of bias; no study was rated as low risk of bias. In prognostic factor studies assessed with QUIPS, the most frequent sources of bias were related to confounding and statistical analysis/reporting, reflecting limited or inconsistent adjustment for key clinicopathologic prognosticators (stage and nodal status-related variables) and variability in analytic transparency and reporting. Information on attrition and follow-up was commonly limited, contributing to moderate risk ratings in the attrition domain in several studies. Outcome measurement was generally considered lower risk, as survival outcomes were typically derived from clinical records or public datasets using standard definitions.

For prognostic model studies assessed with PROBAST, recurrent concerns were concentrated in the analysis domain, reflecting risks of overfitting and optimism (limited external validation), alongside incomplete reporting of model development and validation procedures in some studies. Outcome definitions were generally well specified, whereas predictor definitions and cut-offs varied across studies, affecting comparability.

Reporting quality assessed with REMARK [16] was generally acceptable: all included studies exceeded 55% of REMARK items, most scored above 80%, and four scored between 60% and 75%; the checklist was applied without modifications and scored according to the structure of each study (Table S3). However, it is critical to explicitly contrast these optimistic REMARK scores with the results of the QUIPS and PROBAST assessments. While the majority of studies demonstrated acceptable reporting quality (effectively describing their methods and findings), nearly all remained at a moderate or high risk of bias. This stark contrast underscores that adequate reporting does not equate to methodological rigor; despite being well reported, the underlying study designs, statistical validations, and handling of confounders were frequently flawed or insufficient.

### 3.5. Data Synthesis

Extracted prognostic effect estimates are summarized in Table 3. Across included studies, prognostic associations were derived from survival analyses conducted within TSCC patient cohorts, where gene- or signature-based variables were related to overall survival and/or disease-free outcomes. Many studies first compared tumor and normal tissues to identify differentially expressed candidates; however, prognostic effects were mainly evaluated by stratifying cancer patients (e.g., high vs. low expression or risk score), rather than by tumor–normal contrasts. OS was the most reported endpoint and was therefore used as the primary outcome for structured cross-study comparison. Reported hazard ratios, 95% CIs, sample sizes, and the type of survival modeling (Kaplan–Meier/log-rank, Cox regression; univariable or multivariable) are presented for each biomarker. Most biomarkers were reported in single studies, and effect estimates were derived from heterogeneous cohorts and analytic pipelines; accordingly, comparisons across biomarkers were descriptive rather than inferential.

**Figure 4:**
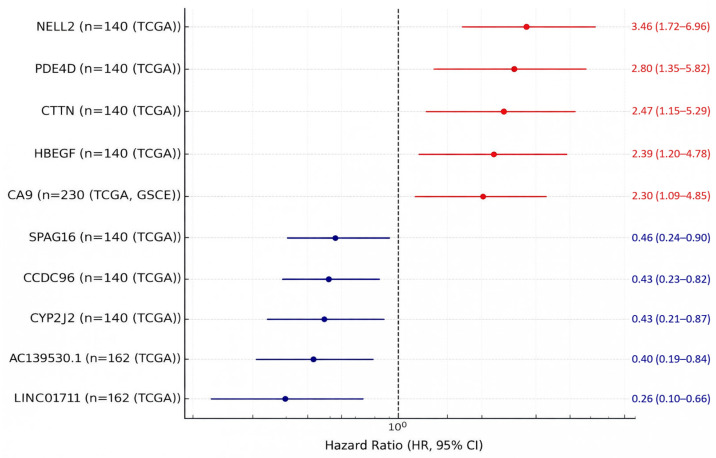
The most statistically robust biomarkers among those with extractable multivariable OS estimates.

Among biomarkers evaluated using multivariable Cox regression for OS, several showed statistically significant associations with increased risk (hazard ratio > 1), including *NELL2*, *PDE4D*, *CTTN*, *HBEGF*, and *CA9* (Figure 4). In contrast, several biomarkers demonstrated statistically significant associations consistent with reduced risk (hazard ratio < 1), including *SPAG16*, *AC139530.1*, *LINC01711*, *CCDC96*, and *CYP2J2* (Figure 4). Biomarkers supported only by univariable analyses are reported in Table 3 for completeness but were not used to define the set of biomarkers supported by adjusted (multivariable) modeling.

*CA9* was the only biomarker reported in more than one included study and supported by multivariable OS analysis, enabling exploratory quantitative pooling. This analysis should be interpreted strictly as exploratory and not as evidence of reproducible clinical effect. In the two eligible studies, a higher *CA9* expression was associated with worse OS: Zhu et al. [28], HR 1.263 (95% CI 1.0957–1.456), and Wang et al. [33], HR 2.30 (95% CI 1.09–4.854). In a fixed-effect model, the pooled association was HR 1.29 (95% CI 1.12–1.48). In a random-effects model, the pooled estimate was HR 1.52 (95% CI 0.88–2.61). Between-study heterogeneity was moderate (Q = 2.39, df = 1, *p* = 0.12; I2 = 58.1%; tau2 = 0.104). Importantly, because this exploratory pooling includes only two studies (k = 2), these calculated heterogeneity statistics (I^2^, τ^2^) and the corresponding random-effects estimate are inherently unstable. Given this statistical limitation, alongside substantial differences in analytical platforms and clinical cohorts, the pooled hazard ratio should be viewed solely as a preliminary signal highlighting the need for standardized prospective validation.

Furthermore, the results of the pathway synthesis are presented in Table 4. Only signaling pathways and functional processes for which biomarkers were evaluated through survival analyses are included, while those reported in a single study were omitted.

### 3.6. Evidence Tiering and Translational Readiness Prioritization

To move beyond a flat catalog of candidates, we applied the prespecified evidence tier framework (Section 2.8) and summarized translation-relevant attributes (endpoint, confounding control, validation strategy, assay feasibility, and clinical decision linkage). While 85 distinct biomarkers were identified across the 17 included studies, most were reported in single studies and could not be meaningfully ranked across heterogeneous platforms and endpoints.

Tier 1 evidence (replicated, adjusted OS, assay-feasible): Only *CA9* met Tier 1 criteria within the eligible evidence base, as it was reported in more than one included study, supported by multivariable OS modeling, and was feasible for routine measurement.

Tier 2 evidence (adjusted OS plus independent validation): A limited subset of candidates met Tier 2 criteria, primarily where multivariable survival modeling was paired with a distinct validation cohort and/or a clinically oriented validation workflow. These candidates represent plausible near-term priorities for validation studies designed around concrete TSCC decisions, but they currently lack replication across multiple eligible studies within the defined inclusion window.

Tier 3–4 evidence (limited validation, univariable/exploratory): The majority of biomarkers fell into Tier 3 or Tier 4 because they were supported only by univariable analyses, relied on internal-only validation (including split-sample analyses of the same public cohort), lacked transparent cut-off justification, or did not report sufficient information to extract comparable effect estimates. Risk-of-bias concerns were most frequently aligned with confounding control and analytic optimism/overfitting, and these limitations were carried into the translational readiness mapping.

It is crucial to emphasize that this tier classification represents a relative prioritization within a currently limited evidence base, heavily reliant on single studies and overlapping public datasets, rather than any indication of clinical readiness or immediate translational applicability. Table 5 provides a concise, clinically oriented summary of the ten statistically most robust prognostic biomarkers identified in this review (based on the most precise survival associations from the extracted OS effect estimates) to improve readability and interpretability in the main text. To ensure transparency and completeness, Supplementary Table S5 presents the full evidence-tiering and translational readiness mapping for all biomarkers and signatures extracted from Table 2 and Table 3, including endpoint(s), whether adjustment was performed (as reported), validation type, assay/specimen, candidate clinical decision linkage, and key limitations.

**Table 5:** Priority biomarkers and translation readiness.

Biomarker/Signature	Evidence Tier (1–4)	Endpoint (OS/DFS/RFS/DSS)	Adjusted? (Y/N; Covariates)	Validation (None/Internal/External/Multi-Cohort)	Specimen + Assay	Potential Clinical Context of Use (Neck/Adjuvant/Surveillance)	Key Limitations (RoB Flags)
CA9	1	OS	Y; (Cox; covariates NR); Y; (multivariable Cox; covariates NR)	Replicated across 2 eligible studies; external (public cohorts: TCGA +GEO)	Microarray; Microarray + NGS (scRNA-seq) (GSE172577)	Adjuvant; surveillance	Cut-off heterogeneity; residual confounding; k = 2
NELL2	2	OS	Y; (multivariable Cox; covariates NR)	External (public cohorts: TCGA + GEO)	NGS (RNA-seq)	Adjuvant; surveillance	Single-study effect; residual confounding
PDE4D	2	OS	Y; Yes (multivariable Cox; covariates NR)	External (public cohorts: TCGA + GEO)	NGS (RNA-seq)	Adjuvant; surveillance	Single-study effect; residual confounding
CTTN	2	OS	Y; (multivariable Cox; covariates NR)	External (public cohorts: TCGA + GEO)	NGS (RNA-seq)	Adjuvant; surveillance	Single-study effect; residual confounding
HBEGF	2	OS	Y; (multivariable Cox; covariates NR)	External (public cohorts: TCGA + GEO)	NGS (RNA-seq)	Adjuvant; surveillance	Single-study effect; residual confounding
AC139530.1	2	OS	Y; (multivariable Cox; covariates NR)	Internal (single public cohort: TCGA)	NGS (RNA-seq)	Surveillance	Single-study effect; residual confounding
LINC01711	3	OS	Y; (multivariable Cox; covariates NR)	Internal (single public cohort: TCGA)	NGS (RNA-seq)	Surveillance	Single-study effect; residual confounding
CCDC96	2	OS	Y; (multivariable Cox; covariates NR)	External (public cohorts: TCGA + GEO)	NGS (RNA-seq)	Surveillance	Single-study effect; residual confounding
CYP2J2	2	OS	Y; (multivariable Cox; covariates NR)	External (public cohorts: TCGA + GEO)	NGS (RNA-seq)	Surveillance	Single-study effect; residual confounding
SPAG16	2	OS	Y; Y; (multivariable Cox; covariates NR)	External (public cohorts: TCGA + GEO)	NGS (RNA-seq)	Surveillance	Single-study effect; residual confounding

## 4. Discussion

TSCC remains the most common malignancy of the oral cavity and is characterized by aggressive clinical behavior and suboptimal long-term survival. In population-based data, five-year relative survival remains modest [34], and outcomes have not improved substantially over recent decades despite advances in surgery, radiotherapy, and systemic therapy [10]. This clinical reality underscores a persistent unmet need: patients classified within the same TNM stage can experience markedly different trajectories, indicating that conventional clinicopathologic staging alone incompletely captures the biological heterogeneity that drives recurrence, metastasis, and survival. Molecular biomarkers derived from high-throughput profiling may help refine risk stratification, inform surveillance intensity, and ultimately support more individualized treatment decisions.

This systematic review identified 17 eligible omics-based studies evaluating prognostic molecular biomarkers in TSCC. Regarding anatomic sub-site specificity, a major methodological strength of this review is our strict focus on oral (mobile) tongue squamous cell carcinoma. Recognizing the distinct biological and HPV-related profiles of the oropharynx, we deliberately excluded base-of-tongue tumors. As detailed in our PRISMA flow diagram, 104 studies were rigorously excluded specifically due to “wrong site”. The evidence base was recent, with most studies published after 2019, reflecting an increased adoption of RNA sequencing, microarray profiling, and integrative bioinformatics pipelines. The geographic distribution was skewed toward East Asia (predominantly China), with fewer studies from other regions, which may limit generalizability. Across studies, discovery workflows most often used transcriptome-wide approaches (RNA-seq and/or microarrays), while targeted laboratory techniques, including RT-qPCR and immunohistochemistry, were commonly used as secondary validation steps. Importantly, immunohistochemistry was not treated as an exclusion criterion when used for validation of omics-discovered candidates, as it served as a confirmatory rather than discovery role.

At the study-design level, the included literature clustered into two major comparison frameworks, each aligned with distinct clinical questions: Cancer vs. cancer phenotype comparisons (within-tumor cohorts): Studies compared TSCC subgroups with different clinical phenotypes, most frequently LNM status or occult nodal disease. These studies aimed to identify biomarkers linked to aggressive biology and metastatic propensity, sometimes extending analyses to survival outcomes (recurrence, OS, DFS). Tumor vs. normal/adjacent tissue comparisons: Studies first identified differentially expressed transcripts between tumor and non-tumor tissues and then linked candidates to prognosis using Kaplan–Meier analyses, Cox regression, and, in some cases, multi-gene risk scores and nomograms. This separation is clinically meaningful: phenotype-based designs are directly anchored to metastatic behavior, while tumor–normal designs prioritize differential expression and often produce broader biomarker signatures that require additional steps for clinical translation.

Across the included studies, prognostic signals converged on a limited set of biological themes despite the heterogeneity in biomarkers, platforms, and analytic workflows. Phenotype-based comparisons (e.g., nodal versus non-nodal cohorts) most often highlighted programs related to immune signaling, cytoskeletal remodeling, and cell motility, consistent with an emphasis on metastatic competence. In contrast, tumor–normal profiling studies more frequently identified broader transcriptional programs linked to microenvironmental adaptation and extracellular matrix remodeling, often operationalized through enrichment results and multi-gene risk models. Taken together, the evidence suggests that TSCC prognosis is shaped by a coordinated dysregulation of tumor–stroma interaction, inflammatory signaling, and adhesion/invasion mechanisms; however, most candidate biomarkers remain single-study findings and should be interpreted cautiously.

Within-cancer comparisons highlighted biomarkers associated with nodal dissemination and/or recurrence-related endpoints. Coding-gene candidates included immune- and invasion-related signals such as *TNFRSF10C* copy-number variation [17], linked to DFS in node-negative patients; *IER3* [18], linked to nodal metastasis and survival endpoints; *ACTA1* [20] (associated with early regional/occult metastasis); and defensin-associated genes [19] *(DEFB4A/DEFB103B/DEFB4B*), showing reduced expression in association with nodal involvement in early-stage disease. These findings collectively support the concept that TSCC metastatic competence may reflect a coordinated dysregulation of immune signaling, cytoskeletal remodeling, and epithelial barrier-associated programs. Non-coding and genomic biomarkers were also represented in phenotype-based designs. *ADAMTS9-AS2* [22] was associated with adverse clinicopathologic characteristics (including LNM) and poorer outcomes, with mechanistic work suggesting a regulatory axis influencing EMT-related behavior. *TERT* promoter mutations (C228T/C250T) [23] were associated with advanced stage and reduced OS, particularly in younger patients, and were supported by a larger clinical validation cohort relative to most omics-only studies. Taken together, phenotype-based studies provide biologically plausible candidates linked to metastatic behavior, but many were constrained by small discovery sample sizes and variable analytic approaches, reinforcing the need for larger, prospectively curated TSCC cohorts.

Tumor–normal studies produced both single-gene candidates and multi-marker models across coding and non-coding classes. Several studies reported lncRNA-based prognostic panels (e.g., autophagy-related [24] and immune-related lncRNA signatures [29]), typically using TCGA-derived training/testing splits and Cox modeling to construct risk scores. A circRNA candidate (*circ_0000919*) [26] was associated with worse OS and correlated with a higher T/N/TNM stage in validation tissue cohorts. These approaches emphasize the utility of non-coding transcriptional programs as prognostic readouts, but they also raise implementation barriers because risk scores require locked assays, standardized normalization, and reproducible cutoffs across laboratories.

TSCC is also shaped by epigenetic dysregulation, including DNA methylation changes, chromatin remodeling, and non-coding RNA-mediated regulation, which can influence transcriptional programs associated with invasion, immune escape, and treatment resistance [35]. Within the eligible evidence base of this review, epigenetic information was captured primarily through transcriptomic readouts (non-coding RNAs) rather than the direct profiling of methylation or chromatin marks. Notably, microRNAs (miRNAs) represent an important regulatory layer because they modulate gene expression post-transcriptionally and can reflect oncogenic pathway activity; however, few of the included omics-based TSCC prognostic studies reported miRNA-centered prognostic models [10]. Where miRNAs appeared, they were typically embedded within broader regulatory frameworks (e.g., ceRNA networks involving lncRNAs/circRNAs) rather than evaluated as standalone prognostic predictors [36].

A central finding of this review is that the TSCC prognostic biomarker literature is highly heterogeneous in cohort definition, geography, molecular platforms, endpoints, and validation strategy—features that materially constrain translation and increase the likelihood of inflated, non-reproducible effects. At the design level, studies clustered into two non-equivalent comparison frameworks—tumor–normal (T vs. C; 10/17, 58.8%) and phenotype-stratified cancer–cancer comparisons (N0 vs. N+; 7/17, 41.2%)—which address different biological questions and therefore cannot be pooled without explicit stratification. The evidence base was geographically skewed, limiting generalizability, while platform heterogeneity (bulk RNA-seq 9/17, microarray 4/17, DNA-based sequencing 2/17, plus TCGA transcriptome-only workflows and scRNA-seq in single studies) and inconsistent endpoints (OS 13/17, DFS 2/17, recurrence-focused endpoints 1/17) further reduced comparability. In addition, a reliance on public datasets was frequent (TCGA 7/17; GEO 4/17), raising overlap risk where discovery and “validation” may not be truly iwith disagreements resolved by condependent; sample sizes varied widely (approximately 19–513, median ~120), which can destabilize multivariable modeling. For these reasons, cross-biomarker comparisons in this review should be interpreted as descriptive rather than inferential, and the term “accuracy” is not appropriate without head-to-head evaluation using comparable discrimination and calibration metrics. Within this heterogeneous landscape, the dominant threats to validity impede reproducibility and provide a coherent explanation for why many biomarkers show promising single-study associations but fail to replicate.

Within those constraints, the most interpretable effect estimates were those derived from multivariable Cox regression for OS because they adjust for major clinicopathologic confounders (e.g., stage, nodal status). In the extracted multivariable OS analyses, several biomarkers showed increased risk (HR > 1), including *NELL2*, *PDE4D*, *CTTN*, *HBEGF*, and *CA9*, while a set of biomarkers showed associations consistent with reduced risk (HR < 1), including *SPAG16*, *AC139530.1*, *LINC01711*, *CCDC96*, and *CYP2J2*. These findings identify candidates that warrant follow-up, but they do not establish a definitive hierarchy of prognostic superiority across biomarkers.

To systematically synthesize our findings and guide future research directions, we introduced an evidence tier framework, categorizing the identified biomarkers based on the robustness of their supporting data (e.g., presence of multivariable survival analysis and cohort validation). Reflecting the scarcity of robust data, only a single biomarker (CA9) met the criteria for Tier 1, while only seven single biomarkers or multi-gene signatures reached Tier 2. While this framework successfully highlights these most promising molecular candidates, its interpretation requires strict caution. The current literature is fundamentally constrained by a lack of true independent validation, with the vast majority of biomarkers supported merely by single studies or by computational replication across overlapping public datasets. Therefore, it must be noted that these tiers establish a relative hierarchy among the available findings. Given the field’s dependence on singular cohorts and overlapping data, a higher tier placement denotes comparative methodological robustness rather than actual readiness for routine clinical implementation.

Notably, *CA9* was the only biomarker identified in more than one included study [28,33], providing a rare instance of marker overlap within this review. While validation across independent cohorts is a prerequisite for clinical translation, CA9’s recurrence in this specific evidence base should be interpreted as an encouraging, albeit preliminary, signal rather than definitive proof of broad reproducibility. *CA9* is a well-established hypoxia-inducible, HIF-1-regulated enzyme that contributes to extracellular acidification and intracellular pH homeostasis, thereby supporting key cancer hallmarks such as metabolic adaptation to hypoxia, invasion, and metastatic potential within an acidic tumor microenvironment [37,38]. In head and neck and oral cavity squamous cell carcinomas, *CA9* overexpression has been associated with aggressive clinicopathologic features and adverse outcomes, consistent with the direction of association observed in the included TSCC studies [39]. These biological links strengthen the plausibility of *CA9* as a prognostic biomarker and support its prioritization for further clinical validation, particularly because it can be assessed using routine pathology workflows. The limited recurrence of *CA9* in the included evidence base likely reflects substantial heterogeneity in platforms, analytic thresholds, and study aims, as well as the restriction to TSCC-specific, high-throughput discovery studies within the 2014–2024 window, rather than suggesting that *CA9* is biologically unimportant. For these exact reasons of methodological and clinical heterogeneity, the exploratory quantitative pooling performed for *CA9* possesses a highly limited practical utility; the pooled hazard ratio serves strictly as an illustrative signal rather than a definitive clinical metric, and it does not resolve the broader reproducibility challenges inherent in this field. In contrast, many other statistically significant associations were single-study observations or were derived from overlapping public cohorts (particularly TCGA), which limits independent confirmation. For example, *TNFAIP3* [28] was highlighted because it was supported by survival analysis and appeared within a recurring pathway theme (IL-17 signaling), but it was not replicated across independent TSCC cohorts in the included omics-based studies; therefore, it should be regarded as a promising but currently single-study candidate.

Across studies, identical genes were rarely replicated, yet there was meaningful convergence at the pathway/program level, suggesting that TSCC prognosis may be governed by a limited number of recurring biological mechanisms rather than isolated markers. Among studies reporting enrichment analyses, recurrent themes included IL-17 signaling, ECM–receptor interaction, and focal adhesion, aligning with inflammatory signaling, extracellular matrix remodeling, adhesion, invasion, and metastatic propensity. We interpreted recurrent pathway signals using an established cancer biology organizing principle (hallmarks-of-cancer framework) and the domain literature on hypoxia, ECM/adhesion-driven invasion, inflammatory/immune signaling, and telomere maintenance to propose hypothesis-generating mechanistic axes for TSCC progression [40,41,42].

Integrating these pathway signals with reported candidates supports four mechanistic axes that plausibly underpin aggressive TSCC behavior and explain cross-study variability in specific marker identities: (i) hypoxia and microenvironmental stress adaptation, consistent with the prognostic association of *CA9* and coherent with selection pressures in poorly perfused tumor regions that promote metabolic rewiring, pH regulation, and treatment resistance; (ii) ECM remodeling, invasion, and focal adhesion signaling, a program that mechanistically supports local invasion and dissemination—central determinants of TSCC prognosis; (iii) inflammatory and immune signaling, including IL-17-related biology, consistent with a model in which immune–tumor interactions and inflammatory microenvironments modulate progression and recurrence risk; and (iv) replicative immortality/genomic maintenance, suggested by DNA-level alterations such as *TERT* promoter mutations, providing a plausible link to sustained proliferation and adverse outcomes in defined patient subsets. Nonetheless, pathway-level conclusions remain hypothesis-generating, as enrichment outputs depend on input gene lists, background sets, analytic choices, and variable reporting across studies.

It is critical to emphasize that the current evidence base for the biomarkers identified in this review is strictly prognostic. Therefore, extending their potential relevance to serve as clinical tools for outcome stratification, anti-tumoral response, treatment sensitivity, or adjuvant decision-making represents a speculative, hypothesis-generating concept. While such applications are biologically plausible—given that many of these markers reflect aggressive tumor biology related to treatment resistance (e.g., hypoxia, extracellular matrix remodeling, and genomic maintenance)—the included studies were not specifically designed to evaluate response to targeted antitumor therapies. Consequently, the clinical relevance of these markers as response-oriented tools remains purely exploratory and will strictly require validation in prospective, treatment-annotated cohorts with standardized response and survival endpoints.

The principal limitation of the available literature, and therefore of this review, is heterogeneity: most biomarkers were evaluated in single studies, study designs and endpoints varied (e.g., OS, DFS, and recurrence-related outcomes), and analytic workflows and biomarker cut-offs were inconsistent. Crucially, the marked heterogeneity in the selection of clinicopathologic covariates (e.g., adjusting for varied combinations of TNM stage, grade, risk scores, or specific clinical habits like smoking) across multivariable models severely restricts direct cross-study comparability and prevents the robust pooling of independent effect sizes. Beyond these inconsistencies, a fundamental limitation persists regarding the clinical depth of the multivariable models themselves. Although our evidence tier framework prioritized biomarkers supported by multivariable survival analyses, these primary models predominantly adjusted only for the aforementioned broad parameters. Aggressive histopathologic features with profound prognostic implications—most notably extracapsular extension, perineural invasion and precise depth of invasion—were rarely, if ever, incorporated. Consequently, it remains largely unclear whether these high-tier molecular candidates provide true incremental prognostic value when evaluated alongside established, high-risk clinical factors. In addition, many studies relied on publicly available datasets and, in some cases, overlapping TCGA-derived cohorts, reducing the independence of evidence across publications and increasing the likelihood of optimistic effect estimates. A prominent example of this involves the two studies identifying *CA9* [28,33], which utilized overlapping public datasets, specifically the TSCC TCGA and GSE31056 cohorts. From a computational perspective, the fact that these independent teams employed entirely distinct bioinformatic pipelines—ferroptosis-related DEG screening versus mixed microarray/scRNA-seq integration—and converged on the same biomarker strongly reinforces the analytical robustness of *CA9*. Nevertheless, it is critical to emphasize that such computational replication does not equate to true biological or clinical validation. Furthermore, from a statistical standpoint, this dataset overlap introduces a critical risk of patient double-counting in our exploratory meta-analysis. This fundamental limitation further underscores why the pooled hazard ratio cannot be treated as a definitive clinical effect size but rather serves strictly as a mathematically illustrative signal. Ultimately, this pervasive reliance on shared public data reflects a broader structural constraint of the entire biomarker research field, rather than a limitation exclusive to this systematic review.

Furthermore, our methodological choices introduce inherent limitations to this systematic review. A residual limitation exists regarding the primary studies reliant on public databases (e.g., TCGA or GEO). Although these specific cohorts were filtered by the original authors to include only “tongue” samples, the accuracy of such subsets fundamentally depends on the primary clinical annotations provided to the repositories. Consequently, the possibility that a small fraction of misclassified base-of-tongue cases inadvertently remained within these public datasets cannot be entirely ruled out, which could theoretically introduce minor biological confounding. In addition, the strict exclusion of non-English publications introduces language bias, potentially omitting relevant prognostic findings published in regional journals. Similarly, the decision to exclude gray literature, while a standard procedural choice to maintain peer-reviewed quality, represents a possible source of publication bias, as studies with negative or null prognostic associations are traditionally less likely to be published in major indexed databases.

Importantly, risk-of-bias assessment using QUIPS and PROBAST indicated that most included studies were at a high risk of bias, mostly driven by incomplete control for confounding in prognostic factor analyses and analysis-related limitations in prognostic model development (including overfitting and limited external validation). This pervasive methodological fragility fundamentally challenges the apparent robustness and credibility of even the most promising biomarkers identified in our tier system. Specifically, the frequent failure to rigorously adjust for key clinicopathologic covariates heavily increases the likelihood of inflated prognostic effect sizes. Therefore, findings should be interpreted as hypothesis-generating rather than definitive for clinical implementation. Accordingly, the direct clinical utility of the current evidence base remains limited, and the reported candidates should not be considered ready for routine prognostic use without further independent validation and the demonstration of incremental value beyond established clinicopathologic predictors. While REMARK assessment suggested generally acceptable reporting completeness, reporting quality does not mitigate the underlying methodological limitations captured by QUIPS/PROBAST.

Future studies should move from discovery-oriented reporting toward a decision-focused validation pipeline explicitly aligned to TSCC management. Priority should be given to converting candidates into reproducible, FFPE-compatible assays with pre-specified cut-offs, followed by truly independent external validation in multicenter, geographically diverse cohorts with transparent handling of missing data and follow-up. Validation models should pre-register a core confounder set (at minimum stage and nodal status, and where available DOI, PNI, LVI, margin status, and treatment) and quantify incremental prognostic value beyond clinicopathologic baselines using discrimination and calibration metrics (and, where feasible, decision curve analysis), rather than relying on *p*-values alone. Ultimately, the translational endpoint is to test whether biomarker-guided strategies improve patient-relevant outcomes through prospective clinical utility studies that evaluate changes in management using standardized endpoints and pre-specified analysis plans.

## 5. Conclusions

This systematic review synthesized omics-driven evidence on prognostic molecular biomarkers in tongue squamous cell carcinoma (TSCC) and identified a broad but highly heterogeneous landscape of candidate markers and signatures. Across 17 eligible studies, most findings were derived from single cohorts using diverse platforms, endpoints, cut-offs, and modeling strategies, with limited genuinely independent external validation and frequent risk-of-bias concerns related to confounding control and analytic optimism. Accordingly, the current evidence base should be interpreted as hypothesis-generating rather than practice-changing.

Within these constraints, only a small subset of candidates provided multivariable overall survival effect estimates, and *CA9* was the only biomarker reported in more than one included study, supporting it as a priority for further investigation. At the pathway level, recurrent themes, including inflammatory signaling and tumor–stroma interaction programs such as ECM–receptor interaction and focal adhesion, suggest mechanistic convergence despite the limited replication of individual genes. Ultimately, given the pervasive methodological heterogeneity and the moderate-to-high risk of bias across the current literature, no single biomarker or multi-gene signature identified in this review is currently ready for routine clinical or prognostic use in TSCC.

## Figures and Tables

**Table 1:** Summary of the PICO framework applied in this systematic review, outlining the study population, intervention, comparators, and primary outcomes assessed.

PICO Element	Systematic Review of Prognostic Molecular Biomarkers in TSCC
P—Population	TSCC patients
I—Intervention	Identification of molecular biomarkers
C—Comparison	Omics-based molecular profiles of TSCC patients, healthy individuals
O—Outcome	Prognostic outcomes (OS, DFS, etc.)

**Table 2:** List of eligible studies. N0 = TSCC without metastatic lymph nodes, N+ = TSCC with metastatic lymph nodes, T—TSCC, C = adjacent normal tissue, NGS = next generation sequencing, ↑ = higher expression or better survival, ↓ = lower expression, poorer survival, KM = Kaplan–Meier, DEG—differentially expressed genes, LNC = Long non-coding RNA.

N0 vs. N+ or T vs. C	Reference	Sample Size	Biomarkers	Method of Biomarker Detection	Biomarker-Associated Pathway	Pathway Sources/Methods	Association with Prognosis	Method of Correlation with Prognosis
N0 vs. N+	Yang X et al./2021/ China [17]	41N0, 19N+	CNVs of gene TNFRSF10C	NGS (WES)	PI3K-Akt signaling pathway, migration	KEGG	↑ TNFRSF10C → ↑ DFS, N0	KM
N0 vs. N+	Xiao F et al./2019/China [18]	14N0, 5N+	IER3 gene	Microarray + NGS (RNA-seq)	PI3K-Akt signaling pathway, MAPK signaling pathway, hypoxia, angiogenesis, lymphangiogenesis, VEGF-C, apoptosis	GSVA	↑ IER3 → poorer OS/DFS, N+	KM, HR(OS)
N0 vs. N+	Lee DY et al./2022/South Korea [19]	35 N0, 12N+	DEFB4A, DEFB103B, and DEFB4 genes	NGS (RNA-seq)	Calcium signaling pathway, muscle contraction	KEGG, GO	↓ DEFB4A/DEFB103B/DEFB4B → N+	Expression/DEGs, cell infiltration
N0 vs. N+	Lee DY et al./2021/ S. Korea [20]	65 N0, 41N+	ACTA1 gene	NGS (RNA-seq)	Muscle contraction (through actin)	KEGG, GO	↑ACTA1 → N+	Expression/limma DEGs
N0 vs. N+	Yang X et al./2017/ China [21]	6N0, 6N+, 12C	MFAP5 and TNNC1 genes	Microarray	Focal adhesion	KEGG, GO	↑ MFAP5 → poor prognosis MFAP5 is an independent prognostic biomarker of occult metastasis MFAP5/TNNC1 → TSCC recurrence	KM, log-rank; Cox
N0 vs. N+	Li et al./2019/ China [22]	41N0, 35N+	LNC ADAMTS9-AS2	Microarray (lncRNA)	Growth/invasion	-	↑ ADAMTS9-AS2 → poor prognosis, ↑ TNM or size, and ↑ clinical stage	KM, log-rank
N0 vs. N+	Kim et al./2023/ S. Korea [23]	16T (<45 years old), 28T (>45 years old)	TERTp mutations-C228T and C250T mutations	NGS (DNA-based)	-	-	In young TSCC patients: ↑ TERTp mutations → ↑ TNM, ↓ OS	KM, log-rank, Cox
T vs. C	Ren Y et al./2023/ China [24]	147T, 15C (TCGA)	Panel of 10 LNC	NGS (RNA-seq)	MAPK signaling pathway	KEGG, GO, GSEA	Score nomogram: ↑ score → ↑ T, ↓ OS and is independent biomarker	KM, Cox
T vs. C	Liu M et al./2021/ China [25]	127T, 13C (TCGA) & 28T (GEO)	Panel of 15 genes	NGS (RNA-seq)	-	-	Score nomogram: ↑ score → ↓ OS and is independent prognostic biomarker	KM, log-rank, Cox
T vs. C	Liu M et al./2022/ China [26]	60T, 60C	circ_0000919	Microarray	MAPK signaling pathway, angiogenesis, lymphangiogenesis, VEGF-C	KEGG, GO	↑ circ_0000919 → ↑ T/N/TNM, ↓ OS	KM, log-rank
T vs. C	Dou H et al./2024/ China [27]	23T, 49C & 62T, 16C (GEO)	Gene SEMA3C	Microarray + NGS (RNA-seq)	Migration, growth/invasion	KEGG, GO	↑ SEMA3C→ ↓ OS	KM, log-rank, Cox
T vs. C	Zhu, H et al./2022/ China [28]	60T, 60N & 217T, 93C (GEO)-143T (TCGA)	Genes CA9, (TNFAIP3 and NRAS)	Microarray	IL-17 signaling pathway, ECM–receptor interaction	GSEA, GSVA-KEGG, GO	↑ CA9 → ↓ OS and is independent prognostic biomarker—TNFAIP3 and NRAS did not reach statistical significance	KM, Cox
T vs. C	Hu et al./2023/ USA [29]	94T, 15C (TCGA)	Panel of 6 LNC	Transcriptome data from TCGA	-	-	The risk model was an important independent indicator of OS → distinguish between high- and low-risk TSCC; patients in the high-risk group → ↓ OS	KM, Cox
T vs. C	Li et al./2019/ China [30]	126T, 13C (TCGA)	hsa-miR-1229-3p, AL359851.1	NGS (RNA-seq)	Cytokine receptor interaction, PI3K-Akt signaling pathway, focal adhesion, MAPK signaling pathway, IL-17 signaling pathway, focal adhesion, calcium signaling pathway	ORA-KEGG, GO	↓ NAGS, hsa-miR-1229-3p, and AL359851.1 → improved outcomes Data divided into two groups: sub-B → ↑ OS	KM, log-rank
T vs. C	Thangaraj et al./2021/ India [31]	100T, 100C	LAMC2, MMP9 and ECAD at ITF, TNC and PDPN	NGS (RNA-seq)	Cytokine receptor interaction, PI3K-Akt signaling pathway, focal adhesion, ECM–receptor interaction	ORA-KEGG, GO GSEA	↑ TNC/PDPN → occult N+ MMP9, LAMC2, DSG2, PLAU, FOXM1 and MYO1B are linked to failure of treatment in the early-stage patients	KM, log-rank, Cox
T vs. C	Jin et al./2021/ China [32]	147T, 15C (TCGA)	PGK1, GPI, and RPE	NGS (RNA-seq)	IL-17 signaling pathway	ssGSEA & GSEA-KEGG, GO	↑ PGK1/GPI/RPE is associated → ↓ OS The risk model was an independent prognostic biomarker	KM, log-rank, Cox
T vs. C	Wang et al./2020/ China [33]	125T, 11C (TCGA), & 23T, 73C (GEO)	CA9	Microarray + NGS (scRNA-seq) (GSE172577)	Calcium signaling pathway	ORA-KEGG, GO	↑ CA9 → ↑ T, ↓ OS and is an independent prognostic biomarker	KM, log-rank, Cox

**Table 3:** Data extracted from the included studies.

Biomarkers	HR (OS)	95% Lower CI	95% Upper CI	Sample Size	Survival Analysis Methods
IER3 *	2.01	1.21	3.36	19	Kaplan–Meier
MFAP5 + TNNC1 *	7.854	1.64	37.621	24	Multivariate
TERTp mutation *	3.003	1.028	8.759	44	Multivariate
AL160006.1	0.6723	0.4529	0.9978	162 (TCGA)	Multivariate
AC139530.1	0.4012	0.1923	0.8372	162 (TCGA)	Multivariate
AL139287.1	1.2553	1.0076	1.564	162 (TCGA)	Multivariate
LINC01711	0.2596	0.1021	0.66	162 (TCGA)	Multivariate
LINC02560	0.9369	0.8897	0.9866	162 (TCGA)	Multivariate
NELL2	3.46	1.72	6.9 6	140 (TCGA)	Multivariate
PDE4D	2.8	1.35	5.82	140 (TCGA)	Multivariate
CCDC96	0.43	0.23	0.82	140 (TCGA)	Multivariate
ADGRG6	2.06	1.01	4.17	140 (TCGA)	Multivariate
CTTN	2.47	1.15	5.29	140 (TCGA)	Multivariate
HBEGF	2.39	1.2	4.78	140 (TCGA)	Multivariate
ADTRP	2.15	1.06	4.35	140 (TCGA)	Multivariate
CYP2J2	0.43	0.21	0.87	140 (TCGA)	Multivariate
RFC4	2.16	1.07	4.36	140 (TCGA)	Multivariate
SPAG16	0.46	0.24	0.9	140 (TCGA)	Multivariate
ABCA4	0.48	0.25	0.92	140 (TCGA)	Multivariate
ITGA3	1.93	1	3.7	140 (TCGA)	Multivariate
circ_0000919	6.687	1.516	29.49	120	Kaplan–Meier/log-rank
SEMA3C	2.284/6.388	1.315/1.595	3.967/25.58	158 (GEO)	Kaplan–Meier/log-rank
TNFAIP3	0.43	0.25	0.76	573 (GEO, TCGA)	Univariate
NRAS	0.47	0.27	0.83	573 (GEO, TCGA)	Univariate
CA9 [28]	1.263	1.0957	1.456	573 (GEO, TCGA)	Multivariate
MIR4713HG	1.610596	1.128103	2.299451	109	Multivariate
AC104088.1	1.402013	1.104656	1.779414	109	Multivariate
AC083967.1	1.787962	1.418558	2.25356	109	Multivariate
FNDC1-IT1	1.552811	1.06642	2.261043	109	Multivariate
MMP9	3.09	1.07	8.9	200	Univariate
LAMC2	2.91	1.36	6.21	200	Univariate
ECAD in ITF	3.11	1.48	6.51	200	Univariate
PGK1	1.00557	1.00232	1.00883	162 (TCGA)	Multivariate
RPE	1.07985	1.011023	1.153363	162 (TCGA)	Multivariate
GPI	1.014747	1.000517	1.02918	162 (TCGA)	Multivariate
CA9 [33]	2.3	1.09	4.854	230 (TCGA, GEO)	Multivariate

* Biomarkers from comparison of cancer tissues with different clinicopathologic phenotypes.

**Table 4:** Results of pathway synthesis. ITF = invasive tumor front.

Pathways	Biomarkers	HR	95% Lower CI	95% Upper CI
Interleukin (IL)-17 signaling pathway	TNFAIP3	0.43	0.25	0.76
	NRAS	0.47	0.27	0.83
	CA9 [28]	1.26	1.096	1.46
ECM-receptor interaction	LAMC2	2.91	1.36	6.21
	ECAD at ITF	3.11	1.48	6.51
	MFAP5 + TNNC1	7.85	1.64	37.62
Focal adhesion	MMP9	3.09	1.07	8.9
	LAMC2	2.91	1.36	6.21
	ECAD at ITF	3.11	1.48	6.51

## Data Availability

No new data were created or analyzed in this study. Data sharing is not applicable to this article.

## Supplementary Materials

The following supporting information can be downloaded at: https://www.mdpi.com/article/10.3390/cimb48040389/s1. References [43,44,45,46,47,48,49,50,51,52,53,54,55,56,57,58,59,60,61,62,63,64,65,66,67,68,69,70,71,72,73] are cited in the supplementary materials.

## Abbreviations

The following abbreviations are used in this manuscript:

TSCC	Tongue squamous cell carcinoma
ASR	Age-standardized incidence rate
SCC	Squamous cell carcinoma
miRNAs	MicroRNAs
IHC	Immunohistochemistry
PRISMA	Preferred Reporting Items for Systematic Reviews and Meta-Analyses
OS	Overall survival
DFS	Disease-free survival
DSS	Disease-specific survival
OSCC	Oral squamous cell carcinoma
IPD	Individual participant data
QUIPS	Quality In Prognosis Studies tool for Risk of Bias
PROBAST	The Prediction model Risk Of Bias assessment tool
REMARK	Reporting Recommendations for Tumor Marker Prognostic Studies
HR	Hazard ratio
CI	Confidence interval
RFS	Recurrence-Free Survival
T	Tumor specimen
C	Control (non-tumor) specimen
TCGA	The Cancer Genome Atlas Project
GEO	Gene Expression Omnibus
lncRNA or LNC	Long non-coding RNA
NGS	Next-generation sequencing
N1-N3, or N+	Tumor specimen with lymph node metastasis
N0	Tumor specimen without lymph node metastasis
KEGG	Kyoto Encyclopedia of Genes and Genomes
DEG	Differentially expressed gene
CNV	Copy number variant
LNM	Lymph node metastasis
GSVA	Gene Set Variation Analysis
GO	Gene Ontology
KM	Kaplan–Meier
Cox	Cox proportional hazards regression
