# Supplementary material for: Omics-Derived Prognostic Biomarkers in Tongue Squamous Cell Carcinoma: A Systematic Review with Risk-of-Bias Appraisal and Translational Prioritization
Source: Curr Issues Mol Biol. 2026 Apr 10;48(4):389. doi: 10.3390/cimb48040389 (PMC13114870; doi:10.3390/cimb48040389)
Supplement: Supplementary file 1 [file cimb-48-00389-s001.zip › cimb-4209090-supplementary.pdf]

Review

Omics-Derived Prognostic Biomarkers in Tongue Squamous Cell Carcinoma: A Systematic Review with Risk-of-Bias Appraisal and Translational Prioritization

Ioannis Astreidis [1], Ilias Kostidis [2], Andigoni Malousi [3], Konstantinos Paraskevopoulos [1,*], Dimitris Andreadis [4], Konstantinos Vahtsevanos [1], Ioannis Vizirianakis [2,5]

[1] Department of Oral & Maxillofacial Surgery, Aristotle University of Thessaloniki, Specialized Cancer Treatment and Reconstraction Centre, General Hospital of Thessaloniki "George Papanikolaou", Thessaloniki, Greece; astrimax23@gmail.com (I.A.); vaxtseva@gmail.com (K.V.)
[2] Laboratory of Pharmacology, School of Pharmacy, Aristotle University of Thessaloniki, Thessaloniki, Greece; kostiilias@pharm.auth.gr (I.K.); ivizir@pharm.auth.gr or vizirianakis.i@unic.cy (I.V.)
[3] Laboratory of Biological Chemistry, School of Medicine, Aristotle University of Thessaloniki, Thessaloniki, Greece; andigoni@auth.gr
[4] Department of Oral Medicine/Pathology, School of Dentistry, Aristotle University of Thessaloniki, Thessaloniki, Greece; dandrea@dent.auth.gr
[5] Department Health Sciences, School of Life & Health Sciences, University of Nicosia, Nicosia, Cyprus
* Correspondence, authors to whom e-mail should be addressed; kosparaskevopoulos@dent.auth.gr

Table S1. QUIPS [1] risk of bias assessment

Study ID	First author	Year	Country	Data/assay	Outcome(s)	Prognostic analysis (reported)	External validation	Study participation	Study attrition	Prognostic factor measurement	Outcome measurement	Study confounding	Statistical analysis/reporting	Overall risk of bias	Notes (basis for judgement)
[2]	Yang X	2021	China	WES, Variants SNV, CNV, TMB MATH aneuploidy score (+ANNOVAR annotation); Tumor tissues and/or blood samples	DFS	Kaplan Meier between groups (+log-rank test, Wilcoxon test, Fisher's exact test)	False	Moderate	Moderate	Moderate	Low	High	High	High	Biomarker(s): CNV status of TNFRSF10C (and other differential CNVs identified by WES/CNV analysis) evaluated in relation to DFS. Discovery cohort n=41 (19 N+ / 22 N0), with TCGA-based validation for association with LNM and survival. Survival comparisons largely based on Kaplan–Meier/log-rank; limited multivariable adjustment reported for prognostic

Study ID	First author	Year	Country	Data/assay	Outcome(s)	Prognostic analysis (reported)	External validation	Study participation	Study attrition	Prognostic factor measurement	Outcome measurement	Study confounding	Statistical analysis /reporting	Overall risk of bias	Notes (basis for judgement)
															inference.
[3]	Xiao F	2019	China	Public transcriptomic datasets (GSE2280 microarray: primary TSCC vs metastatic lymph node) with pathway analysis (e.g., GSVA) and validation using TCGA TSCC expression and clinical data; additional functional validation in TSCC cell line (Tca-8113) as reported.	OS	Kaplan Meier between groups high/low biomarker (+t-test, ANOVA, Kaplan–Meier analysis, log-rank test)	False	Moderate	Moderate	Moderate	Low	High	High	High	Biomarker(s): they concluded the key gene was gene IER3. Validation n (if reported): 148.0.; QUIPS rationale: secondary analysis of GEO/TCGA; survival analyses largely unadjusted; hub-gene selection and testing within same datasets.
[4]	Lee DY	2022	South Korea	mRNA sequencing, DEGs, analysis of immune cell infiltration, pathway analyses,; tumor tissues (Fresh-frozen)		Using the Student t-test, DEG expression was compared between two groups. (+ fold-change, adjusted P-values)	False	Moderate	Moderate	Low	Low	High	Moderate	High	Biomarker(s): genes DEFB4A, , DEFB103B, , DEFB4B. Validation n (if reported): nan.; QUIPS rationale: small selected early-stage cohort; group comparisons without adjustment for key clinicopathologic confounders; no survival modelling.
[5]	Lee DY	2021	South Korea	the Linear Models for Microarray Data (Limma)package in R, differentially expressed genes (DEGs); tissue cancer FFPE	Occult lymph node metastasis / early regional metastasis (nodal status;	Differential expression (limma) and group comparisons (t-tests); no survival modelling; no multivariable adjustment	True	High	Moderate	Moderate	Low	High	High	High	TCGA discovery compared non-equivalent clinical groups (T1/2 N2/3 vs T4 N0), introducing selection bias and confounding; authors note potential selection bias and lack of survival correlation. Prognostic factor (ACTA1/actin-associated gene expression) measured via

Study ID	First author	Year	Country	Data/assay	Outcome(s)	Prognostic analysis (reported)	External validation	Study participation	Study attrition	Prognostic factor measurement	Outcome measurement	Study confounding	Statistical analysis/reporting	Overall risk of bias	Notes (basis for judgement)
					cN0/pN0 vs cN0/pN+)										RNA-seq and validated in independent FFPE cohort by IF/IHC. Outcome (occult nodal metastasis) based on pathological nodal status, but analyses relied on group comparisons without multivariable adjustment.
[6]	Yang, X.	2017	China	Affymetrix HTA2.0 high-density oligonucleotide (transcriptome) array, RT-PCR, immunohistochemistry; Fresh-frozen tumor tissue and matched normal mucosa from TSCC patients	DFS (metastasis-/recurrence-free), OS	Kaplan–Meier survival analysis (log-rank), chi-square tests for associations, and univariable/multivariable Cox regression for prognostic assessment.	False	Moderate	Moderate	Low	Low	Moderate	High	Moderate	Single-centre T2 TSCC cohorts; discovery microarray (n=12) with validation by qRT-PCR (n=32) and IHC (n=61). IHC scoring performed by blinded assessors; cut-off defined as IRS>0. Multivariable Cox reported but based on small sample/event counts and limited covariate adjustment; wide CIs.
[7]	Li, Y.	2019	China	Human LncRNA Expression Microarray (Arraystar v3.0), qRT-PCR, Western blotting, Boyden chamber migration and invasion assays, luciferase reporter assays, RNA immunoprecipitation (RIP) using AGO2, in situ hybridization (ISH), and cellular fractionation using the PARIS Kit, bioinformatics (DIANA tools, TargetScan,	OS	Student's t-test, one-way ANOVA, Spearman's correlation, Kaplan–Meier survival analysis, log-rank test (assessed via Kaplan-Meier survival analysis and log-rank test)	False	Moderate	Moderate	Moderate	Moderate	High	High	High	Retrospective single-centre cohort (n≈76; 41 N0/35 N+) with lncRNA microarray and qRT-PCR/functional validation. Prognosis assessed using Kaplan–Meier/log-rank without multivariable adjustment for key confounders (e.g., stage/nodal status/treatment). Cut-off definition and missing data handling not fully reported.

Study ID	First author	Year	Country	Data/assay	Outcome(s)	Prognostic analysis (reported)	External validation	Study participation	Study attrition	Prognostic factor measurement	Outcome measurement	Study confounding	Statistical analysis/reporting	Overall risk of bias	Notes (basis for judgement)
				MiRanda); TSCC tissue, adjacent non-tumor tissue and normal human oral keratinocytes (NOKs)											
[8]	Kim, Sehui	2023	South Korea	Next-generation sequencing (NGS) using the TruSight Oncology 500 assay, and Sanger sequencing for validation, PCR, and Nirvana, and IGV; Formalin-fixed, FFPE and frozen tumor tissue blocks	OS	Chi-square test, Fisher's exact test, t-test, Kaplan–Meier analysis, log-rank test, and Cox proportional hazards regression (univariate and multivariate) (IGV visualization, and manual review of mutations)	False	Moderate	Moderate	Low	Low	Moderate	Moderate	Moderate	Retrospective clinical sequencing cohort of advanced OTSCC (NGS panel; n=44) with additional validation cohort (n=298). Mutation status is objectively measured; survival endpoints defined and analysed with Kaplan–Meier and Cox (univariable/multivariable). Selection limited to clinically sequenced advanced cases and covariate set for adjustment not fully specified.
[9]	Liu H	2022	China	circRNA microarray screening in 5 paired TSCC and control tissues; circRNA–miRNA prediction (e.g., TargetScan/miRanda); RT-qPCR validation in 60 paired TSCC/control tissues with clinicopathologic correlation analyses.	OS	RT-qPCR was performed to determine the relative expression levels of 10 candidate circRNAs (top 5 upreg and top 5 downgrulated circs in tumor and paired Ctrl tissues derived from 60 patients with TSCC. - - - The correlation analysis between tumor circRNA expression and clinical features was carried out	False	Moderate	Moderate	Low	Low	High	High	High	Single-centre cohort (validation n=60 TSCC with paired controls) following microarray screening (5 pairs). OS analysed by Kaplan–Meier/log-rank using median expression split; no multivariable adjustment for clinicopathologic confounders. Follow-up and missing data handling not reported in detail.

Study ID	First author	Year	Country	Data/assay	Outcome(s)	Prognostic analysis (reported)	External validation	Study participation	Study attrition	Prognostic factor measurement	Outcome measurement	Study confounding	Statistical analysis /reporting	Overall risk of bias	Notes (basis for judgement)
						using the Spearman's rank correlation test. A Kaplan-Meier curve was used to display the OS. According to the median level of circRNA expression, the latter was divided into circRNA high and low expression. The association between the expression levels of circRNAs in the tumor samples and OS was evaluated using the log-rank test.									
[10]	Dou H	2024	China	Used limma to identify differentially expressed genes; functional enrichment; WGCNA/LASSO/ random forest feature selection; public GEO datasets with tumour and normal tongue tissues; plus downstream validation/experiments as reported.	OS	The Kaplan–Meier and log-rank tests are extensively employed in survival analysis. (Unpaired t-test, Kaplan–Meier analysis, log-rank test, Cox proportional hazards model)	False	Moderate	Moderate	Moderate	Low	High	High	High	Public-dataset driven identification of SEMA3C with survival evaluated in GEO cohorts by median split and Cox HR estimation. No clear multivariable adjustment for key clinical confounders; high risk of optimism due to data-driven feature selection and survival testing within overlapping datasets; limited reporting of missing data/follow-up.
[11]	Thangaraj, Soundara	2021	India	RNA sequencing (RNA-seq) for discovery and qPCR and	OS	Differential expression with FDR correction; correlation	True	Moderate	Moderate	Moderate	Low	High	High	High	Biomarker(s): genes LAMC2, VIM, HIF1A, TWIST2, SOX2, TNC, PDPN, MMP9, DSG2,

Study ID	First author	Year	Country	Data/assay	Outcome(s)	Prognostic analysis (reported)	External validation	Study participation	Study attrition	Prognostic factor measurement	Outcome measurement	Study confounding	Statistical analysis /reporting	Overall risk of bias	Notes (basis for judgement)
	Viveka			immunohistochemistry (IHC) for validation; primary tongue cancer tissues and corresponding apparently uninvolved adjacent tissues preserved in RNAlater and formalin-fixed paraffin-embedded blocks		analyses; Kaplan–Meier survival analysis and log-rank testing; Cox regression as reported in the primary study.									PLAU, FOXM1, MYO1B and ECAD (E-cadherin). Validation n (if reported): nan. RoB rationale: Single-centre design with small RNA-seq discovery set and larger validation cohort; outcomes (OS/DFS/recurrence) derived from clinical follow-up. Prognostic associations mainly based on Kaplan–Meier/log-rank and/or univariable Cox for biomarkers, with limited adjustment for key confounders (e.g., stage/nodal status) in biomarker-specific analyses; multiple testing and data-driven marker selection increase risk of bias.
[12]	Wang, Shuang	2020	China	RNA sequencing (mRNA-seq from TCGA) and microarray (GSE31056, Affymetrix platform), Real-time RT-PCR, Immunohistochemistry, differential expression analysis; OTSCC tissue samples and adjacent non-tumor tissues from TCGA and GEO for CA9	OS	Kaplan-Meier survival analysis, log-rank test, Cox proportional hazards model, Student's t-test, and Spearman correlation (Differential gene expression was analyzed using the limma package in R, followed by GO/KEGG enrichment via DAVID, PPI network construction with STRING, and	True	Moderate	Moderate	Low	Low	Moderate	Moderate	Moderate	Biomarker(s): carbonic anhydrase 9 (CA9) is a protein. Validation n (if reported): 50.0. RoB rationale: Retrospective analyses using TCGA/GEO transcriptomic data with an independent small institutional validation set (RT-qPCR/Western blot/IHC). OS assessment standard; multivariable Cox models reported with clinicopathologic covariates, but cut-off selection and overlapping public datasets may introduce

Study ID	First author	Year	Country	Data/assay	Outcome(s)	Prognostic analysis (reported)	External validation	Study participation	Study attrition	Prognostic factor measurement	Outcome measurement	Study confounding	Statistical analysis /reporting	Overall risk of bias	Notes (basis for judgement)
						clustering via Cytoscape/MCODE)									optimism/selection bias.

Table S2. PROBAST [13] risk of bias assessment

Study ID	First author	Year	Country	Data/assay	Outcome(s)	Model development/validation (reported)	External validation	Nomogram reported	Predictors	Participants	Outcome	Analysis	Overall risk of bias	Notes (basis for judgement)
[14]	Ren Y	2023	China	TCGA TSCC RNA-seq: identification of autophagy-related lncRNAs; Cox regression-based signature construction; risk score calculation; model performance assessed with ROC/C-index and calibration; nomogram developed integrating clinical variables.	OS	kaplan maeier, cox regression multi kai uni varuate analysis, nomogram (+Pearson correlation, univariate Cox regression, multivariate Cox regression, Kaplan–Meier survival analysis, ROC curves, Wilcoxon test, ANOVA)	False	Yes	Moderate	Moderate	Low	High	High	Retrospective TCGA-only development with limited/unclear external validation; predictor selection and coefficient estimation performed in same dataset; missing data handling and event-per-variable considerations not clearly reported.
[15]	Liu M	2021	China	TCGA RNA-seq (tumour vs normal) with differential expression; LASSO and Cox regression to build 15-gene signature; internal training/testing split; nomogram including clinical factors; performance assessed by ROC/C-index/calibration; external validation in GEO cohort (n=28).	OS	Differential expression and feature selection (including LASSO) to derive a 15-gene prognostic signature; risk score construction; Kaplan–Meier and Cox regression; performance evaluation with C-index, calibration plots and time-dependent ROC; nomogram integrating clinical variables; external validation in a GEO	True	Yes	Moderate	Moderate	Low	Moderate	Moderate	Model includes internal validation and an external GEO cohort; however, feature selection is data-driven and event counts/missing data handling are not fully transparent, creating residual risk of overfitting/optimism.

Study ID	First author	Year	Country	Data/assay	Outcome(s)	Model development/validation (reported)	External validation	Nomogram reported	Predictors	Participants	Outcome	Analysis	Overall risk of bias	Notes (basis for judgement)
						cohort (n=28) reported.								
[16]	Zhu, H.	2022	China	Integrated analysis of TSCC transcriptomic datasets (GEO/TCGA) with differential expression and ferroptosis-related gene filtering; survival modelling (Cox) and risk-score/signature development; ROC/AUC evaluation; CA9 protein validation by immunohistochemistry in an independent tissue set.	OS	Univariable and multivariable Cox regression for candidate genes; construction of a ferroptosis-related prognostic signature/risk score; time-dependent ROC/AUC reported; validation reported in an independent cohort/dataset; IHC validation for CA9.	True	No	Moderate	Moderate	Low	High	High	Model derived via data-driven feature selection across public datasets; although multivariable Cox and ROC/AUC are reported and CA9 is validated by IHC, details on handling of missing data, event counts, and measures to limit overfitting (e.g., bootstrapping) are limited; external validation sample(s) are relatively small/heterogeneous.
[17]	Hu, Daniel	2023	USA	Transcriptome data from TCGA, immune gene data from ImmPort, edgeR for differential expression; TSCC tumor tissues and adjacent normal tissues (TCGA)	OS	edgeR, univariate and multivariate Cox regression, Kaplan–Meier survival, ROC analysis, PCA and Pearson correlation to identify immune-related lncRNAs (Principal Component Analysis (PCA) and differential expression analysis with edgeR)	False	No	Moderate	Moderate	Low	High	High	TCGA-derived immune-related lncRNA risk score with internal split only; no independent external validation cohort; potential overfitting and limited reporting on missing data and calibration.
[18]	Li, Zaiye	2019	China	RNA sequencing profiles and miRNA profiling (NGS-based TCGA data using Illumina HiSeq RNASeq and miRNASeq platforms), GEO database, DESeq2 package in R, clusterprofiler in R, STRING website, survival package in R, Starbase v3.0,	OS	DESeq2 package of R, Pearson correlation analysis, chi-square, Kaplan-Meier, log-rank test (Protein-protein interaction (PPI) network construction using STRING and Cytoscape, ceRNA network construction)	True	No	High	Moderate	Low	High	High	Extensive data-driven screening (DEGs, survival KM screening, ceRNA network and consensus clustering) with multiple testing; no locked model coefficients/cutoffs for clinical prediction and limited performance evaluation/calibration.

Study ID	First author	Year	Country	Data/assay	Outcome(s)	Model development/validation (reported)	External validation	Nomogram reported	Predictors	Participants	Outcome	Analysis	Overall risk of bias	Notes (basis for judgement)
				Consensus Cluster Plus package, GEO2R; TSCC tissues and matched normal tissues										
[19]	Jin, Yi	2021	China	RNA sequencing (NGS-based TCGA data), ssGSEA algorithm, consensus clustering, ESTIMATE, CIBERSORT; TSCC tumor and adjacent normal tissues (TCGA database)	OS	Kaplan-Meier survival analysis, univariate and multivariate Cox regression,LASSO Cox regression analysis, ROC analysis, Pearson correlation, and Mann–Whitney U test (STRING website and Cytoscape,with downstream DEG analysis using edgeR)	False	No	Moderate	Moderate	Low	High	High	Predictor class: Coding. Validation n (if reported): nan. RoB rationale: Risk model derived from TCGA expression data using ssGSEA-based clustering and data-driven predictor selection (e.g., LASSO/Cox). Validation appears internal only (no independent external cohort); handling of missing data and events-per-variable not clearly reported, and multiple selection steps raise overfitting/optimism concerns.

Table S3. Table of quality assessment, REMARK [20].

Columns explanation

1. **States the marker examined, the study objectives, and any pre-specified hypotheses.**
2. **Describes the characteristics (e.g., disease stage or co-morbidities) of the study patients, including their source and inclusion and exclusion criteria.**
3. **Describes treatments received and how chosen (e.g., randomized or rule-based).**
4. **Describes types of biological material used (including control samples) and methods of preservation and storage.**

5. **Specifies the assay method used and provide (or reference) a detailed protocol, including specific reagents or kits used, quality control procedures, reproducibility assessments, quantitation methods, and scoring and reporting protocols. Specify whether and how assays were performed blinded to the study endpoint.**
6. **States the method of case selection, including whether prospective or retrospective and whether stratification or matching (e.g., by stage of disease or age) was used. Specifies the time period from which cases were taken, the end of the follow-up period, and the median follow-up time.**
7. **Precisely defines all clinical endpoints examined.**
8. **Lists all candidate variables initially examined or considered for inclusion in models.**
9. **Give rationale for sample size; if the study was designed to detect a specified effect size, give the target power and effect size.**
10. **Specifies all statistical methods, including details of any variable selection procedures and other model-building issues, how model assumptions were verified, and how missing data were handled.**
11. **Clarifies how marker values were handled in the analyses; if relevant, describe methods used for cutpoint determination.**
12. **Describes the flow of patients through the study, including the number of patients included in each stage of the analysis (a diagram may be helpful) and reasons for dropout. Specifically, both overall and for each subgroup extensively examined report the numbers of patients and the number of events.**
13. **Reports distributions of basic demographic characteristics (at least age and sex), standard (disease-specific) prognostic variables, and tumor marker, including numbers of missing values.**
14. **Shows the relation of the marker to standard prognostic variables.**
15. **Presents univariable analyses showing the relation between the marker and outcome, with the estimated effect (e.g., hazard ratio and survival probability).**
16. **For key multivariable analyses, reports estimated effects (e.g., hazard ratio) with confidence intervals for the marker and, at least for the final model, all other variables in the model.**
17. **Among reported results, provides estimated effects with confidence intervals from an analysis in which the marker and standard prognostic variables are included, regardless of their statistical significance.**
18. **If done, reports results of further investigations, such as checking assumptions, sensitivity analyses, and internal validation.**
19. **Interprets the results in the context of the pre-specified hypotheses and other relevant studies; includes a discussion of limitations of the study.**
20. **Discusses implications for future research and clinical value.**

**Article**	**1**	**2**	**3**	**4**	**5**	**6**	**7**	**8**	**9**	**10**	**11**	**12**	**13**	**14**	**15**	**16**	**17**	**18**	**19**	**20**	%YES
[2]	YES	YES	YES	YES	YES	YES	YES	YES	NO	YES	YES	YES	YES	YES	YES	NO	NO	YES	YES	NO	80
[3]	YES	YES	YES	YES	YES	YES	YES	YES	NO	YES	NO	YES	NO	YES	YES	NO	NO	NO	YES	NO	60
[4]	YES	YES	YES	YES	YES	YES	YES	YES	NO	YES	NO	YES	YES	YES	NO	NO	NO	YES	YES	NO	70
[5]	YES	YES	YES	YES	YES	YES	YES	YES	NO	YES	NO	YES	YES	YES	NO	NO	NO	YES	YES	YES	75
[6]	YES	YES	YES	YES	YES	YES	YES	YES	NO	YES	NO	YES	YES	YES	YES	YES	YES	YES	YES	YES	90
[7]	YES	YES	YES	YES	YES	NO	YES	YES	NO	YES	NO	YES	YES	YES	YES	NO	NO	NO	NO	YES	65
[8]	YES	YES	YES	YES	YES	YES	YES	YES	NO	YES	YES	YES	YES	YES	YES	YES	YES	YES	YES	YES	95
[14]	YES	YES	YES	YES	YES	YES	YES	YES	NO	YES	YES	YES	YES	YES	YES	YES	YES	YES	YES	YES	95
[15]	YES	YES	YES	YES	YES	YES	YES	YES	YES	YES	YES	YES	YES	YES	YES	YES	YES	YES	YES	YES	100
[9]	YES	YES	YES	YES	YES	YES	YES	YES	YES	YES	YES	YES	YES	YES	YES	YES	NO	NO	YES	NO	85
[10]	YES	YES	YES	YES	YES	YES	YES	YES	NO	YES	YES	YES	YES	NO	YES	YES	NO	YES	YES	NO	80
[16]	YES	YES	YES	YES	YES	YES	YES	YES	NO	YES	YES	YES	YES	YES	YES	YES	YES	YES	YES	YES	95
[17]	YES	YES	YES	YES	YES	YES	YES	YES	NO	YES	YES	YES	YES	YES	YES	YES	YES	YES	YES	YES	95
[18]	YES	YES	YES	YES	YES	YES	YES	YES	NO	YES	YES	YES	YES	YES	YES	NO	NO	YES	YES	YES	85
[11]	YES	YES	YES	YES	YES	YES	YES	YES	NO	YES	YES	YES	YES	YES	YES	YES	YES	YES	YES	YES	95
[19]	YES	YES	YES	YES	YES	YES	YES	YES	NO	YES	YES	YES	YES	YES	YES	YES	YES	YES	YES	YES	95
[12]	YES	YES	YES	YES	YES	YES	YES	YES	NO	YES	YES	YES	YES	YES	YES	YES	YES	YES	YES	YES	95

(TITLE-ABS-KEY(tscc* OR "tongue squamous cell carcinoma*" otscc* OR "tongue cancer" OR "tongue tumor" OR "carcinoma of the oral tongue" OR "tongue carcinoma" OR "squamous cell carcinoma of the tongue" OR "carcinoma of the tongue")) AND (TITLE-ABS-KEY(prognos* OR survival OR outcome OR "tumor progression" OR signature* OR perineural OR lymphovascular OR clinic* OR "lymph node" OR "extranodal extension" OR vascular OR metastasis OR invasion OR "pathological grade" OR "TNM stage" OR size OR recurrence)) AND (TITLE-ABS-KEY(biomarker* OR inhibit* OR "long noncoding RNA*" OR "long non-coding RNA*" OR lncrna* OR "tumor suppressor gene*" OR microrna* OR mirna* OR "epithelial mesenchymal transition" OR emt OR "competitive endogenous RNA*" OR cerna* OR circrna* OR "circular RNA*" OR indicator* OR mir* OR mrna OR upregulat* OR downregulat* OR up-regulat* OR down-regulat* OR deregulat* OR prognosticator*)) AND NOT (DOCTYPE (ed) OR DOCTYPE (ma) OR DOCTYPE (ip) OR DOCTYPE (re) OR DOCTYPE (re) OR DOCTYPE (re) OR DOCTYPE (sr) OR DOCTYPE (tr)) AND (EXCLUDE (DOCTYPE , "tb") OR EXCLUDE (DOCTYPE , "le") OR EXCLUDE (DOCTYPE , "no") OR EXCLUDE (DOCTYPE , "er") OR EXCLUDE (DOCTYPE , "sh") OR EXCLUDE (DOCTYPE , "cp")) AND (LIMIT-TO (PUBYEAR , 2014) OR LIMIT-TO (PUBYEAR , 2015) OR LIMIT-TO (PUBYEAR , 2016) OR LIMIT-TO (PUBYEAR , 2017) OR LIMIT-TO (PUBYEAR , 2018) OR LIMIT-TO (PUBYEAR , 2019) OR LIMIT-TO (PUBYEAR , 2020) OR LIMIT-TO (PUBYEAR , 2021) OR LIMIT-TO (PUBYEAR , 2022) OR LIMIT-TO (PUBYEAR , 2023) OR LIMIT-TO (PUBYEAR , 2024)) AND (LIMIT-TO (LANGUAGE , "English"))

Figure S1.. Scopus search strategy applied for the systematic review, combining terms for TSCC, prognosis, and biomarkers (final search performed on 22 July 2024)

((TI=(TSCC* OR "tongue squamous cell carcinoma*" OR OTSCC* OR "tongue cancer" OR "tongue tumor" OR "carcinoma of the oral tongue" OR "tongue carcinoma" OR "squamous cell carcinoma of the tongue" OR "carcinoma of the tongue")
OR AB=(TSCC* OR "tongue squamous cell carcinoma*" OR OTSCC* OR "tongue cancer" OR "tongue tumor" OR "carcinoma of the oral tongue" OR "tongue carcinoma" OR "squamous cell carcinoma of the tongue" OR "carcinoma of the tongue"))
AND
(TI=(prognos* OR survival OR outcome OR "tumor progression" OR signature* OR perineural OR lymphovascular OR clinic* OR "lymph node" OR "extranodal extension" OR vascular OR metastasis OR invasion OR "pathological grade" OR "TNM stage" OR size OR recurrence)
OR AB=(prognos* OR survival OR outcome OR "tumor progression" OR signature* OR perineural OR lymphovascular OR clinic* OR "lymph node" OR "extranodal extension" OR vascular OR metastasis OR invasion OR "pathological grade" OR "TNM stage" OR size OR recurrence))
AND
(TI=(biomarker* OR inhibit* OR "long noncoding RNA*" OR "long non-coding RNA*" OR lncrna* OR "tumor suppressor gene*" OR microrna* OR mirna* OR "epithelial mesenchymal transition" OR emt OR "competitive endogenous RNA*" OR cerna* OR circrna* OR "circular RNA*" OR indicator* OR mir* OR mrna OR upregulat* OR downregulat* OR up-regulat* OR down-regulat* OR deregulat* OR prognosticator*)
OR AB=(biomarker* OR inhibit* OR "long noncoding RNA*" OR "long non-coding RNA*" OR lncrna* OR "tumor suppressor gene*" OR microrna* OR mirna* OR "epithelial mesenchymal transition" OR emt OR "competitive endogenous RNA*" OR cerna* OR circrna* OR "circular RNA*" OR indicator* OR mir* OR mrna OR upregulat* OR downregulat* OR up-regulat* OR down-regulat* OR deregulat* OR prognosticator*)))
NOT DT=(editorial OR "meta-analysis" OR preprint OR review OR "retracted publication" OR "retraction of publication" OR "systematic review" OR "technical report" OR Erratum OR "Meeting Abstract" OR "Early Access" OR Correction OR Letter OR "Proceeding Paper" OR Retraction OR "Editorial Material" OR "Publication With Expression Of Concern")
AND LA=(English)
AND DOP=(2014-2024)

Figure S2. Web of Science search strategy applied for the systematic review, combining terms for TSCC, prognosis, and biomarkers (final search performed on 22 July 2024)

#1 (TSCC* OR tongue NEXT squamous NEXT cell NEXT carcinoma* OR OTSCC* OR "tongue cancer" OR "tongue tumor" OR "carcinoma of the oral tongue" OR "tongue carcinoma" OR "squamous cell carcinoma of the tongue" OR "carcinoma of the tongue"):ti,ab
#2 (prognos* OR survival OR outcome OR "tumor progression" OR signature* OR perineural OR lymphovascular OR clinic* OR "lymph node" OR "extranodal extension" OR vascular OR metastasis OR invasion OR "pathological grade" OR "TNM stage" OR size OR recurrence):ti,ab
#3 (biomarker* OR inhibit* OR long NEXT noncoding NEXT RNA* OR lncRNA* OR tumor NEXT suppressor NEXT gene* OR microRNA* OR miRNA* OR "epithelial-mesenchymal transition" OR EMT OR competitive NEXT endogenous NEXT RNA* OR ceRNA* OR circRNA* OR circular RNA* OR indicator* OR miR* OR mRNA OR upregulat* OR downregulat* OR up-regulat* OR down-regulat* OR deregulat* OR prognosticator*):ti,ab
#4 #1 AND #2 AND #3 AND ("journal article" OR "Book or thesis"):pt AND english:la with Cochrane Library publication date Between Jan 2014 and Dec 2024

Figure S3. Cochrane search strategy applied for the systematic review, combining terms for TSCC, prognosis, and biomarkers (final search performed on 22 July 2024)

Table S4. Table for data charting

Study ID	Sample size (cases)	Sample size (controls)	Technology / platform	Specimen type	Biomarker(s)	Pathway / biological process	Validation sample size (cases)	Validation sample size (controls)	Prognostic association	How the prognostic conclusion was derived	Study conclusion	Cancer vs cancer comparison	Biomarker class	Nomogram
[2]	19 T1–T2N+ (LNM group), NSNDrink	22 T1–T2N0 (non-LNM group), NSNDrink	WES (whole exome sequencing), Variants SNV, CNV, TMB MATH aneuploidy score (+ANNOVAR annotation)	Tumor tissues and/or blood samples	156 genes with differential CNVs; one of these was TNFRSF10C.	KEGG analysis revealed that leukocyte transendothelial migration and cytokine-cytokine receptor interaction, the main immune-related enriched pathways, were significantly related to LNM as well as the DFS of patients.	411 TCGA head and neck cancer cases with available LNM and OS data; TNFRSF10C CNVs assessed.	133 N+, 100 N0, 178 other (lymph node status not specified).	Cases were stratified by TNFRSF10C CNV status and nodal status; higher TNFRSF10C CNVs were associated with longer DFS in N0 patients.	Kaplan–Meier survival analysis between groups (log-rank), plus Wilcoxon and Fisher's exact tests.	the enhancement of TNFRSF10C CNV was found to have a significant correlation with DFS in tongue cancer patients with non-LNM. - - - the CNV log2 ratio>-0.163 of TNFRSF10C could potentially serve as an important marker for a good prognosis in tongue cancer patients with non-LNM after cervical lymphadenectomy.	Yes	Coding	No
[3]	Primary TSCC tumors vs lymph node metastases (sample sizes not specified), GEO GSE22		analyzing the GSE2280 RNA microarray dataset and the TCGA tongue cancer RNA-seq dataset.	In GEO, tongue tumor samples were compared with metastatic lymph node samples.	Identified IER3 as a key gene.	GSVA-based pathway analysis (KEGG/Hallmark enrichment) and functional assays; IER3 linked to PI3K/AKT activation.	148 cases from TCGA.		we found that patients with high expression of IER3 had poor prognosis (Fig. 3a), - --- tongue cancer patients with lymph node metastasis showed significantly higher expression of IER3 (+assessed via Kaplan–Meier survival analysis and TCGA expression data)	Group comparisons (t-test/ANOVA) and Kaplan–Meier survival analysis (high vs low expression; log-rank).	we found that the hub gene IER3 might predict prognosis tongue cancer, and our in vitro experiments demonstrated that IER3 might promote the progression and lymph node metastasis in tongue cancer, which might be a potential	Yes	Coding	No

	80.										therapeutic target.			
[4]	23 T1T2N 0 pN0	12 T1T2N0 pN+	mRNA sequencing, DEGs, analysis of immune cell infiltration, pathway analyses,	tumor tissues (Fresh-frozen)	genes DEFB4A, , DEFB103B, , DEFB4B	GO/KEGG enrichment analysis (table provided in the article) using DAVID; implicated pathways included chemotaxis, inflammatory response, and antimicrobial defense.	TCGA 39N0	TCGA 31N+	In our study, reduced expression of defensin-related genes (DEFB4A, DEFB103B, and DEFB4B) was associated with regional metastasis to the neck in early-stage tongue cancer. - - - - - -In the TCGA database, DEFB4A and DEFB103B were more highly expressed in the N0 group than in the N+ group, although this difference did not reach statistical significance (Fig. 4).	Using the Student t-test, DEG expression was compared between two groups. (+ fold-change, adjusted P-values)	In conclusion, defensin (DEFB4A, DEFB103B, DEFB4B) may be a novel biomarker for early regional metastasis in T1/2 tongue cancer. However, because the sample size of this study was very small and the differential expression of the genes did not reach significance in the TCGA dataset, further validation should be performed to establish the clinical usefulness of these findings.	Yes	Coding	No
[5]	group 1:T1/2 and N2/3 (n = 41), 2)	group 2: T4 and N0 (n = 65)	the Linear Models for Microarray Data (Limma)package in R, differentially expressed genes (DEGs)	tissue cancer FFPE	genes ACTA1, ACTC1, MYBPC1, DES	Cytoskeleton-related GO/KEGG pathways (e.g., muscle contraction and keratinization) identified using DAVID and KOBAS 3.0.	group 1: T1/2 andN2/3 (n = 36),	group 2: T4 and N0 (n = 25)	ACTA1 gene was shown to be significant up-regulation inpatient with occult metastasis, assessed using mRNA expression analysis via qRT-PCR and protein localization via immunofluorescence and immunohistochemistry.	Two-tailed Student's t-test	In conclusion, genes associated with cytoskeleton,especially ACTA1, is over-expressed in tongue cancerwith early regional metastasis. The gene can be the sur-rogate or adjunctive marker for selecting elective neckdissection of clinically N0 tongue cancer	Yes	Coding	No

											patients			
[6]	12, with or without cervical lymph node metastasis (CLNM) (n = 6 in each group	12	Affymetrix HTA2.0 high-density oligonucleotide (transcriptome) array, RT-PCR, immunohistochemistry	Fresh-frozen tumor tissue and matched normal mucosa from TSCC patients	genes MFAP5 and TNNC1	GO functional analysis and the KEGG pathway analysis indicated the pathways related to tight junction, focal adhesion, cell adhesion and cell-matrix adhesion	32 patients for RT-PCR + 61 patients for immunohistochemistry validation	Matched normal mucosa from the same patients was used as control (32 + 61)	the mRNA and protein levels of MFAP5 were significantly elevated in patients with poor prognosis, thus indicating that MFAP5 could be an independent prognostic marker for TSCC and its occult cervical metastasis & Our results showed that MFAP5 and TNNC1 expressions were associated with CLNM and cervical lymphatic recurrence, assessed using microarray analysis, qRT-PCR, and immunohistochemistry	Kaplan–Meier method, chi-square (chi-square) analysis, Mantel–Haenszel log-rank test, and Cox regression analyses (Affymetrix microarray profiling).	MFAP5 and TNNC1 may be potential markers for predicting occult cervical lymphatic metastasis and prognosis of oral tongue carcinoma	Yes	Coding	No
[7]	41 tumors, N0.	35 tumors, N+.	Human LncRNA Expression Microarray (Arraystar v3.0), qRT-PCR, Western blotting, Boyden chamber migration and invasion assays, luciferase reporter assays, RNA immunoprecipitation (RIP) using AGO2, in situ hybridization (ISH), and cellular fractionation	TSCC tissue, adjacent non-tumor tissue and normal human oral keratinocytes (NOKs)	lncRNA ADAMTS9-AS2	ADAMTS9-AS2 functions as a ceRNA regulating the miR-600/EZH2 axis to promote TSCC cell migration and invasion via the EMT process. Also, EZH2 could be regulated by a new integrated pathway of ADAMTS9-AS2/miR-600.And ADAMTS9-AS2 may promote TSCC growth and metastasis	same cohort	same cohort	high ADAMTS9-AS2 expression was associated with poor prognosis and was closely correlated with tumor size, clinical stage and lymphnode metastasis rate, assessed via Kaplan-Meier survival analysis and log-rank test	Student's t-test, one-way ANOVA, Spearman's correlation, Kaplan–Meier survival analysis, log-rank test (assessed via Kaplan-Meier survival analysis and log-rank test)	ADAMTS9-AS2 is extremely up-regulated in TSCC tissues, with especially high levels of expression in TSCC tissues with lymphnode metastasis, and it plays a cancer-pro-moting role in TSCC proliferation and metastasis. So it could be an independent prognostic factor for TSCC patients with lymphnodemetastasis, and may be a useful tool for predicting TSCC	Yes	Non-coding	No

			using the PARIS Kit, bioinformatics (DIANA tools, TargetScan, MiRanda)			byregulating EZH2 controlled target gene (revealed through microarray analysis, qRT-PCR, luciferase reporter assay, AGO2-dependent RNA immunoprecipitation (RIP), and ISH, confirming their functional regulatory relationship in TSCC progression)					metastasis			
[8]	16 (<45years old)	28 (>45years old)	Next-generation sequencing (NGS) using the TruSight Oncology 500 assay, and Sanger sequencing for validation, PCR, and Nirvana, and IGV	Formalin-fixed, FFPE and frozen tumor tissue blocks	TERT promoter (TERTp) mutations, specifically C228T and C250T (also TP53, CDKN2A, FAT1, NOTCH1, EGFR and CDKN2A mutation)	TERT pathway related to telomerase activation, identified through NGS, visual inspection in IGV, and Sanger sequencing validation	298 (96 young patients)	202 old	TERTp mutations were related to higher TNM stage and poor OS in young OTSCC patients (HR = 3.0; P = 0.044), suggesting that TERTp mutation may serve as a prognostic biomarker for OTSCC in young patients, assessed via Kaplan–Meier survival analysis, log-rank test, and Cox proportional hazards regression	Chi-square test, Fisher's exact test, t-test, Kaplan–Meier analysis, log-rank test, and Cox proportional hazards regression (univariate and multivariate) (IGV visualization, and manual review of mutations)	In conclusion, our study demonstrates that young patients had more frequent TERTp mutations than older patients in advanced OTSCC. Furthermore, TERTp mutations were related to higher stage and worse OS than wild type. So this study suggest that TERTp mutation may serve as a prognostic biomarker for OTSCC in young patients and that	Yes	Non-coding	No

											young OTSCC patients with TERTp mutation may require more aggressive treatment approaches			
[14]	147 TCGA tumors (same cohort as above); 134 retained for analysis.	15 normal	Combined lncRNAs with autophagy-related genes from TCGA data (HTSeq FPKM); co-expression network analysis (Cytoscape).	tongue tumors and adjacent normal tissues from TCGA	10 lncRNAs; LINC02560, AC092747.4, AL139287.1, LIN01711, MIR503HG, AC010326.3, AL160006.1, AL122010.1, AC139530.1, AC009318.2	GO analysis results show that the co-expression network is enriched in biological processes (BP), cellular components (CC) and molecular functions (MF). D KEGG pathway analysis results display that there are abundant signal pathways including Autophagy, Pl3k-Akt signaling pathway, Protein processing in endoplasmic reticulum, MAPK signaling pathway and so on related to co-expressed expression network - egine kai GSEA (+ p53, mTOR, NOD-	-	-	We can conclude from univariate and multivariate Cox regression analysis that autophagy-related lncRNA prognostic signature is an independent prognostic factor significantly related to OS. - the patients with low-risk scores have significantly longer OS times than high-risk scores.In different clinical categories (such as gender, age, grade, AJCC stage, T stage, and N stage), autophagy-related lncRNA prognostic signatures can accurately predict the survival outcome of the different groups, indicating that this prognostic model is accurate and reliable. - - the autophagy-related lncRNA risk score is related to the T stage of TSCC patients.	risk scoring model - --- - kaplan maeier, cox regression multi kai uni varuate analysis, nomogram (+Pearson correlation, univariate Cox regression, multivariate Cox regression, Kaplan–Meier survival analysis, ROC curves, Wilcoxon test, ANOVA)	we identified ten lncRNAs related to autophagy and constructed a lncRNA-mRNA co-expression network to evaluate their functions. The terms and pathways related to autophagy have been significantly enriched ($p < 0.05$) in the functional analysis of GO, KEGG, and GSEA. It implies that autophagy plays a pivotal role in the progression of TSCC and might have a potential as a therapeutic target, which is consistent with previous studies	No	Non-coding	Yes

						like receptor, Toll-like receptor pathways)								
[15]	127 ffromT CGA	13	Differential expression analysis with oncogenic signature enrichment; patients stratified into high- and low-risk groups and OS compared (RNA-seq).	tongue cancer and normal tissues	a 15-gene prognostic signature involving ADTRP, ITGA3, RFC4, CCDC96, CYP2J2, NELL2, SPHK1, SPAG16, HBEGF, S100A9, EGFL6, ADGRG6, PDE4D, ABCA4, and CTTN	(Oncogenic signatures identified via Metascape enrichment analysis; specific pathways are not detailed in the main text)	28 from GEO		Kaplan-Meier survival analysis indicated the high-risk group had significantly poorer overall survival than the low-risk group (p < 0.0001) - - --- - The results indicated that the predictive ability of the 15-gene signature was independent of other clinicopathological factors for overall survival of M0 OTSCC patients (+Univariate Cox regression (bootstrap), LASSO regression, multivariate Cox regression, log-rank test, ROC AUC, C-index, calibration plots)	A prognostic nomogram was developed and evaluated in both training and validation cohorts (calibration/ROC-based assessment).	In conclusion, our results demonstrate our 15-gene signature was independently associated with overall survival in non-distant metastatic OTSCC. Moreover, the prognostic nomogram integrating the 15-gene signature and clinicopathologic al factors has potential to be developed as a prognostic tool.	No	Coding	Yes
[9]	5/60 patients with TSCC	5/60 paired Ctrl tissues	Microarray analysis in five paired TSCC and control tissues; differential expression analysis identified DEcircRNAs; GO/KEGG enrichment and miRanda were used to predict target miRNAs.	tongue cancer and normal tissues	circ_0000919 as prognostic (circRNAs: circ_0020048, circ_0000919, circ_0004525, circ_0002113, circ_0004029, circ_0004503, circ_0008752,	GO and KEGG enrichment analysis demonstrated that these DEcircRNAs were enriched in tumor-associated biological processes, including 'regulation of angiogenesis' and the 'canonical Wnt signaling	60 patients with TSCC	60 paired Ctrl tissues	The higher expression levels of circ_0000919 in tumor tissues were associated with decreased OS time in patients with TSCC (Fig. 6B; P=0.041), and The expression levels of circ_0000919 exhibited a positive association with T stage (P=0.022), N stage (P<0.001) and TNM stage (P<0.001).	RT-qPCR was performed to determine the relative expression levels of 10 candidate circRNAs (top 5 upreg and top 5 downgrulated circs in tumor and paired Ctrl tissues derived from 60 patients with TSCC. - - - The correlation analysis between tumor circRNA expression and	Among the 10 candidate DEcircRNAs, the expression levels of circ_0000919 were increased in TSCC tumor tissues compared with in the Ctrl tissues and associated with higher TNM stage and low OS time of patients with TSCC, suggesting that circ_0000919 could be used as	No	Non-coding	No

					circ_0002300 and circ_0001811.The expression levels of five DEcircRNAs (circ_0004503, circ_0008752, circ_0002300, circ_0020048 and circ_0000919) were associated with pathological grade or tumor clinical stage. Notably, only the expression levels of one DEcircRNA (circ_0000919) were associated with decreased OS)	pathway', as well as in several oncogenic signaling pathways, such as the 'Wnt signaling pathway', 'MAPK signaling pathway' and 'Ras signaling pathway'				clinical features was carried out using the Spearman's rank correlation test. A Kaplan-Meier curve was used to display the OS. According to the median level of circRNA expression, the latter was divided into circRNA high and low expression. The association between the expression levels of circRNAs in the tumor samples and OS was evaluated using the log-rank test.	a diagnostic and prognostic biomarker in TSCC.			
[10]	Two GEO datasets with 23 and 62 tumor samples.	49 and 24 controls, plus 16 normal samples.	limma was used to identify differentially expressed genes; functional enrichment analyses (GO/KEGG)	Tumoral tissues and peritumoral normal tongue tissue	gene SEMA3C	Growth, proliferation, migration, and invasion pathways (e.g., JAK–STAT, NF-kB, and stemness-related processes)	Three GEO datasets for validation; additional validation in 9 paired tongue cancer samples.	9	the results showed that high expression of SEMA3C was a poor prognostic factor in TSCC ($P < 0.05$) (Fig. 5a, b) (assessed via Kaplan–Meier curves and Cox regression)	The Kaplan–Meier and log-rank tests are extensively employed in survival analysis. (Unpaired t-test, Kaplan–Meier analysis, log-rank test, Cox	In this study, we preliminarily screened the key marker gene of TSCC and revealed for the first time that SEMA3C is involved in the development of	No	Coding	No

			and machine-learning tools (e.g., WGCNA, LASSO, random forest), plus in vitro assays (colony formation, wound healing, transwell).			identified via GO analysis and correlation-based enrichment (DAVID).				proportional hazards model)	TSCC. We demonstrated that SEMA3C deletion inhibits TSCC cells growth, migration, and invasion in vitro. Thus, SEMA3C might serve as an early clinical marker for TSCC.			
[16]	60 TSCC tumor tissue samples, 217 tumor samples from 4 GEO datasets used for gene expression analysis, 143 tongue cancer samples from TCGA for survival analysis	60 matched adjacent normal tissue samples, 93 normal samples from GEO datasets for expression analysis	Microarrays, bioinformatics analysis (including GEO, TCGA, FerrDB), and immunohistochemistry (IHC)	TSCC tumor and adjacent normal tissues	ferroptosis-related genes CA9, TNFAIP3 and NRAS	extracellular matrix (ECM)-receptor and interleukin (IL)-17 signaling pathways were identified via KEGG and GO enrichment analysis (clusterProfiler) and GSEA, GSVA analyses			CA9 , TNFAIP3 and NRAS higher expression of these genes indicating poorer prognosis. This was assessed using Cox regression, Kaplan–Meier, ROC analysis, and IHC	Kaplan–Meier analysis, Receiver operating characteristic (ROC) analysis, Univariate and multivariate Cox regression (GSEA, GSVA, PPI network (STRING + Cytoscape), DESeq2 for DEG detection, and visualization with ggplot2 and volcano plots and IHC for protein validation)	Ferroptosis-related genes CA9, TNFAIP3 and NRAS as potential prognostic markers through the ECM-receptor interaction and IL-17 signaling pathways	No	Coding	No
[17]	94 for training (145 TSCC cases were used	15 normal control tissues	Transcriptome data from TCGA, immune gene data from ImmPort, edgeR for	TSCC tumor tissues and adjacent normal tissues (TCGA)	six immune-related signature lncRNAs; lncRNAs MIR4713HG,	from literature	51 for testing	-	The six-lncRNA model was an important indicator of survival rate while it can be used to distinguish TSCC patients between	edgeR, univariate and multivariate Cox regression, Kaplan–Meier survival, ROC analysis, PCA and Pearson	This six-lncRNA prognostic model has clinical significance and may be helpful in the development of personalized	No	Non-coding	No

	from TCGA)		differential expression		AC104088.1, LINC00534, NAALADL2-AS2, AC083967.1, FNDC1-IT1				high- and low-risk, assessed with Kaplan–Meier, Cox regression, and ROC analysis	correlation to identify immune-related lncRNAs (Principal Component Analysis (PCA) and differential expression analysis with edgeR)	immunotherapy strategies			
[18]	126	13	RNA sequencing profiles and miRNA profiling (NGS-based TCGA data using Illumina HiSeq RNASeq and miRNASeq platforms), GEO database, DESeq2 package in R, clusterprofiler in R, STRING website, survival package in R, Starbase v3.0, Consensus Cluster Plus package, GEO2R	TSCC tissues and matched normal tissues	ceRNA:: SLC16A1, E2F7, SCN8A, ZIC2, NR4A1, hsa-miR-337-3p - a total of 15 prognostic DEmRNAs (SLC16A1, E2F7, SCN8A, ZIC2, NR4A1, CLU, GFPT2, CEP55, PLK1, SYBU, STC2, DEPDC1B, MICB, LIFR, BIRC5), 1 DEmiRNA (hsa-miR-337-3p), and 2 DElncRNAs (AC017048.3, AC156455.1) were involved in the ceRNA network -	Cytokine-cytokine receptor interaction, PI3K-Akt signaling pathway, Human papillomavirus infection, Focal adhesion, Calcium signaling pathway, MAPK signaling pathway, cAMP signaling pathway, chemical carcinogenesis, nicotine addiction, IL-17 signaling pathway, and various metabolic and epidermal development processes, identified through GO and KEGG enrichment analyses using the	Validation performed using GEO2R and related approaches; validation focused on biomarker expression rather than prognosis.	-	several genes with differential expressions from tumor and normal tissues were significantly associated with overall survival, assessed using integrated analysis (via Kaplan-Meier method, log-rank test)	DESeq2 package of R, Pearson correlation analysis, chi-square, Kaplan-Meier, log-rank test (Protein-protein interaction (PPI) network construction using STRING and Cytoscape, ceRNA network construction)	The study identified key prognostic biomarkers and two molecular subtypes of TSCC, contributing to potential targeted therapy and improved prognosis prediction	No	Both	No

					hub genes with the highest degree including ALB, FN1, EGF, MM9, KNG1, COL1A1, SPP1, ACTN2	ClusterProfiler R package							
[11]	100	100	RNA sequencing (RNA-seq) for discovery and qPCR and immunohistochemistry (IHC) for validation	primary tongue cancer tissues and corresponding apparently uninvolved adjacent tissues preserved in RNAlater and formalin-fixed paraffin-embedded blocks	genes MMP9, LAMC2, DSG2, PLAU, FOXM1, MYO1B, TNC, PDPN (and ECAD (E-cadherin)->IH)- .	Cytokine-cytokine receptor interaction, Focal adhesion, ECM-receptor interaction and PI3K-Akt signaling pathway were identified using RNA-seq data quantified via FPKM normalization, followed by differential gene expression analysis, and analyzed through GO and KEGG enrichment, GSEA, and PPI network construction using tools like STRING, DAVID, or Cytoscape	same cohort	-	predicting risk of disease relapse and death in OTSCC patients confirmed the expression of LAMC2, MMP9 and ECAD at ITF as the important prognostic indicators. Up-regulation of Tenacin C (TNC) and Podoplanin (PDPN) was significantly correlated with occult node positivity, using Kaplan-Meier survival curves - - --- -- g overexpression namely, MMP9 (P value = 0.02), LAMC2 (P value = 0.02), DSG2 (P value = 0.02), PLAU (P value = 0.02), FOXM1 (P value = 0.02) and MYO1B (P value = 0.02) to be associated with failure of treatment in the early stage patients	Student's t-test, FDR correction, Pearson correlation, Kaplan–Meier survival analysis, Log-rank test, (Transcriptome sequencing, GO and KEGG enrichment analysis via DAVID, STRING-based PPI network, Cytoscape, qRT-PCR (2^−ΔΔCt), and immunohistochemistry (IHC) for biomarker validation, Cellular fractionation)	this cohort study addressed the occult node metastasis prediction with 2 molecular markers and identified a panel of 6 molecular markers that can be used to differentiate the early stage tumors at higher risk of recurrence.	No	Coding	No
[19]	147 TSCC samples	15 paracancerous samples	RNA sequencing (NGS-based TCGA data),	TSCC tumor and adjacent normal	hub genes PGK1, GPI, and RPE among 15	GO and KEGG analysis indicated that	-	-	High expression of PGK1, GPI, and RPE correlates with immune infiltration	Kaplan-Meier survival analysis, univariate and multivariate Cox	15-gene immune signature, immune- related genes and	No	Coding	No

			ssGSEA algorithm, consensus clustering, ESTIMATE, CIBERSORT	tissues (TCGA database)	regulators	some immune-related pathways, such as primary immunodeficiency, Th17 cell differentiation, and Th1/Th2 cell differentiation, were enriched, while GSEA analysis indicated signaling pathways, including interferon-gamma response, hypoxia and glycolysis (the three hubs -> involved in the glycolysis pathway)			and worse overall survival, suggesting prognostic potential, assessed via Kaplan-Meier survival analysis, univariate and multivariate Cox regression	regression,LASSO Cox regression analysis, ROC analysis, Pearson correlation, and Mann–Whitney U test (STRING website and Cytoscape,with downstream DEG analysis using edgeR)	clusters in TSCC, have the potential to guide prognosis and individualized treatments			
[12]	125 TCGA (11) + 23 OTSCC samples from GEO	11 TCGA + 73 from GEO	RNA sequencing (mRNA-seq from TCGA) and microarray (GSE31056, Affymetrix platform), Real-time RT-PCR, Immunohistochemistry, differential expression analysis	OTSCC tumor tissues and adjacent non-tumor tissues from TCGA and GEO (CA9).	carbonic anhydrase 9 (CA9) is a protein	neuroactive ligand-receptor interaction, calcium signaling pathway and transcriptional misregulation: identified using KEGG and GO via DAVID	50 for immunohistochemistry	50 for immunohistochemistry	CA9 is a potential prognostic factor in OTSCC and the expression of CA9 is significantly correlated with the pathological T-stage of OTSCC, assessed via immunohistochemistry, Cox proportional hazards model multivariate analysis, and Kaplan–Meier survival analysis	Kaplan-Meier survival analysis, log-rank test, Cox proportional hazards model, Student’s t-test, and Spearman correlation (Differential gene expression was analyzed using the limma package in R, followed by GO/KEGG enrichment via DAVID, PPI network construction with STRING, and clustering via Cytoscape/MCO	The CA9 gene has the potential to serve as a biomarker and therapeutic target for precise diagnosis and prognostic evaluation of OTSCC	No	Coding	No

										DE)				

Table S5. Evidence tiering and translational readiness mapping for all biomarkers/signatures extracted from Tables 2 and 3.

Abbreviations: OS, overall survival; DFS, disease-free survival; TCGA, The Cancer Genome Atlas; GEO, Gene Expression Omnibus; KM, Kaplan-Meier; HR, hazard ratio; CI, confidence interval; NR, not reported.

Biomarker / signature	Evidence tier	Endpoint(s)	Adjusted?	Validation	Specimen + assay	Clinical decision point(s)	Effect estimate(s)	Study reference(s)	Key limitations
CA9	Tier 1	OS	Yes (Cox; covariates NR); Yes (multivariable Cox; covariates NR)	External (public cohorts: TCGA + GEO)	Microarray; Microarray + NGS (scRNA-seq) (GSE172577)	Adjuvant intensity; surveillance stratification	HR 1.263 (95% CI 1.0957-1.456); HR 2.3 (95% CI 1.09-4.854)	Wang et al./2020/ China[12]; Zhu, H et al./2022/ China[16]	Confounding adjustment/cut-off details often NR; cohort/platform heterogeneity; limited independent prospective validation
15-gene signature score<br>ADTRP<br>ITGA3<br>RFC4<br>CCDC96<br>CYP2J2<br>NELL2<br>SPHK1<br>SPAG16<br>HBEGF<br>S100A9<br>EGFL6<br>ADGRG6<br>PDE4D<br>ABCA4<br>CTTN	Tier 2	OS	Yes (Cox; covariates NR)	External (public cohorts: TCGA + GEO)	NGS (RNA-seq)	Adjuvant intensity; surveillance stratification	NR	Liu M et al./2021/ China[15]	Confounding adjustment/cut-off details often NR; cohort/platform heterogeneity; limited independent prospective validation

10-lncRNA signature score LINC02560, AC092747.4, AL139287.1, LIN01711, MIR503HG, AC010326.3, AL160006.1, AL122010.1, AC139530.1, AC009318.2	Tier 2	OS	Yes (Cox; covariates NR)	Internal (single public cohort: TCGA)	NGS (RNA-seq)	Adjuvant intensity; surveillance stratification	NR	Ren Y et al./2023/ China[14]	Confounding adjustment/cut-off details often NR; cohort/platform heterogeneity; limited independent prospective validation
MFAP5 + TNNC1	Tier 2	OS	Yes (multivariable Cox; covariates NR)	Internal (single clinical cohort)	Microarray	Adjuvant intensity; surveillance stratification	HR 7.854 (95% CI 1.64-37.621)	Yang X et al./2017/ China[6]	Confounding adjustment/cut-off details often NR; cohort/platform heterogeneity; limited independent prospective validation
PGK1	Tier 2	OS	Yes (Cox; covariates NR); Yes (multivariable Cox; covariates NR)	Internal (single public cohort: TCGA)	NGS (RNA-seq)	Adjuvant intensity; surveillance stratification	HR 1.00557 (95% CI 1.00232-1.00883)	Jin et al./2021/ China[19];	Confounding adjustment/cut-off details often NR; cohort/platform heterogeneity; limited independent prospective validation
GPI	Tier 2	OS	Yes (Cox; covariates NR); Yes (multivariable Cox; covariates NR)	Internal (single public cohort: TCGA)	NGS (RNA-seq)	Adjuvant intensity; surveillance stratification	HR 1.014747 (95% CI 1.000517-1.02918)	Jin et al./2021/ China[19];	Confounding adjustment/cut-off details often NR; cohort/platform heterogeneity; limited independent prospective validation
RPE	Tier 2	OS	Yes (Cox; covariates NR); Yes (multivariable Cox; covariates NR)	Internal (single public cohort: TCGA)	NGS (RNA-seq)	Adjuvant intensity; surveillance stratification	HR 1.07985 (95% CI 1.011023-1.153363)	Jin et al./2021/ China[19]	Confounding adjustment/cut-off details often NR; cohort/platform heterogeneity; limited independent prospective validation
6-lncRNA immune signature score MIR4713HG,	Tier 2	OS	Yes (Cox; covariates NR)	Internal (single public cohort: TCGA)	Transcriptome data from TCGA	Adjuvant intensity; surveillance stratification	NR	Hu et al./2023/ USA[17]	Confounding adjustment/cut-off details often NR;

AC104088.1, LINC00534, NAALADL2-AS2, AC083967.1, FNDC1-IT1									cohort/platform heterogeneity; limited independent prospective validation
ECAD in ITF	Tier 3	OS	No (univariable/KM)	Internal (single clinical cohort)	NGS (RNA-seq)	Adjuvant intensity; surveillance stratification	HR 3.11 (95% CI 1.48-6.51)	Thangaraj et al./2021/ India[11]	Confounding adjustment/cut-off details often NR; cohort/platform heterogeneity; limited independent prospective validation
IER3	Tier 3	OS; OS; DFS	NR (HR reported; covariates NR); No (univariable/KM)	Internal (single clinical cohort)	Microarray + NGS (RNA-seq)	Adjuvant intensity; surveillance stratification; Neck management (cN0 risk/occult metastasis)	HR 2.01 (95% CI 1.21-3.36)	Xiao F et al./2019/China/ [3]	Confounding adjustment/cut-off details often NR; cohort/platform heterogeneity; limited independent prospective validation
LAMC2	Tier 3	Nodal metastasis / TNM association (survival NR); OS	No (univariable/KM); Yes (Cox; covariates NR)	Internal (single clinical cohort)	NGS (RNA-seq)	Adjuvant intensity; surveillance stratification; Neck management (cN0 risk/occult metastasis)	HR 2.91 (95% CI 1.36-6.21)	Thangaraj et al./2021/ India[11]	Confounding adjustment/cut-off details often NR; cohort/platform heterogeneity; limited independent prospective validation
MMP9	Tier 3	Nodal metastasis / TNM association (survival NR); OS	No (univariable/KM); Yes (Cox; covariates NR)	Internal (single clinical cohort)	NGS (RNA-seq)	Adjuvant intensity; surveillance stratification; Neck management (cN0 risk/occult metastasis)	HR 3.09 (95% CI 1.07-8.9)	Thangaraj et al./2021/ India[11]	Confounding adjustment/cut-off details often NR; cohort/platform heterogeneity; limited independent prospective validation
NRAS	Tier 3	OS	No (univariable/KM); Yes (Cox; covariates NR)	External (public cohorts: TCGA + GEO)	Microarray	Adjuvant intensity; surveillance stratification	HR 0.47 (95% CI 0.27-0.83)	Zhu, H et al./2022/ China[16]	Confounding adjustment/cut-off details often NR; cohort/platform heterogeneity; limited independent prospective validation
PDPN	Tier 3	Nodal metastasis / TNM association	Yes (Cox; covariates NR)	Internal (single clinical cohort)	NGS (RNA-seq)	Neck management (cN0 risk/occult	NR	Thangaraj et al./2021/ India[11]	Confounding adjustment/cut-off

		(survival NR)				metastasis)			details often NR; cohort/platform heterogeneity; limited independent prospective validation
SEMA3C	Tier 3	OS	No (univariable/KM); Yes (Cox; covariates NR)	Internal (single clinical cohort)	Microarray + NGS (RNA-seq)	Adjuvant intensity; surveillance stratification	HR 2.284 / 6.388 (95% CI 1.315 / 1.595-3.967 / 25.58)	Dou H Liu M et al./2024/ China[10]	Confounding adjustment/cut-off details often NR; cohort/platform heterogeneity; limited independent prospective validation
TERTp mutation	Tier 3	OS	Yes (multivariable Cox; covariates NR)	Internal (single clinical cohort)	NGS (DNA-based)	Adjuvant intensity; surveillance stratification	HR 3.003 (95% CI 1.028-8.759)	Kim, et al./2023/ S. Korea[8]	Confounding adjustment/cut-off details often NR; cohort/platform heterogeneity; limited independent prospective validation
TERTp mutation (C228T/C250T)	Tier 3	OS	Yes (Cox; covariates NR)	Internal (single clinical cohort)	NGS (DNA-based)	Adjuvant intensity; surveillance stratification	NR	Kim, et al./2023/ S. Korea[8]	Confounding adjustment/cut-off details often NR; cohort/platform heterogeneity; limited independent prospective validation
TNC	Tier 3	Nodal metastasis / TNM association (survival NR)	Yes (Cox; covariates NR)	Internal (single clinical cohort)	NGS (RNA-seq)	Neck management (cN0 risk/occult metastasis)	NR	Thangaraj et al./2021/ India[11]	Confounding adjustment/cut-off details often NR; cohort/platform heterogeneity; limited independent prospective validation
TNFAIP3	Tier 3	OS	No (univariable/KM); Yes (Cox; covariates NR)	External (public cohorts: TCGA + GEO)	Microarray	Adjuvant intensity; surveillance stratification	HR 0.43 (95% CI 0.25-0.76)	Zhu, H et al./2022/ China[16]	Confounding adjustment/cut-off details often NR; cohort/platform heterogeneity; limited independent prospective validation

circ_0000919	Tier 3	OS	No (KM/association); No (univariable/KM)	Internal (single clinical cohort)	Microarray	Adjuvant intensity; surveillance stratification	HR 6.687 (95% CI 1.516-29.49)	Liu M et al./2022/ China[9]	Confounding adjustment/cut-off details often NR; cohort/platform heterogeneity; limited independent prospective validation
ACTA1	Tier 4	Nodal metastasis / TNM association (survival NR)	No (KM/association)	Internal (single clinical cohort)	NGS (RNA-seq)	Neck management (cN0 risk/occult metastasis)	NR	Lee DY et al./2021/ S. Korea[5]	Confounding adjustment/cut-off details often NR; cohort/platform heterogeneity; limited independent prospective validation
ADAMTS9-AS2	Tier 4	OS	No (KM/association)	Internal (single clinical cohort)	Microarray (lncRNA)	Adjuvant intensity; surveillance stratification	NR	Li et al./2019/ China[7]	Confounding adjustment/cut-off details often NR; cohort/platform heterogeneity; limited independent prospective validation
AL359851.1	Tier 4	OS	No (KM/association)	Internal (single public cohort: TCGA)	NGS (RNA-seq)	Adjuvant intensity; surveillance stratification	NR	Li et al./2019/ China[18]	Confounding adjustment/cut-off details often NR; cohort/platform heterogeneity; limited independent prospective validation
DEFB103B	Tier 4	Nodal metastasis / TNM association (survival NR)	No (KM/association)	Internal (single clinical cohort)	NGS (RNA-seq)	Neck management (cN0 risk/occult metastasis)	NR	Lee DY et al./2022/South Korea/ [4]	Confounding adjustment/cut-off details often NR; cohort/platform heterogeneity; limited independent prospective validation
DEFB4	Tier 4	Nodal metastasis / TNM association (survival NR)	No (KM/association)	Internal (single clinical cohort)	NGS (RNA-seq)	Neck management (cN0 risk/occult metastasis)	NR	Lee DY et al./2022/South Korea/ [4]	Confounding adjustment/cut-off details often NR; cohort/platform heterogeneity; limited independent prospective

									validation
DEFB4A	Tier 4	Nodal metastasis / TNM association (survival NR)	No (KM/association)	Internal (single clinical cohort)	NGS (RNA-seq)	Neck management (cN0 risk/occult metastasis)	NR	Lee DY et al./2022/South Korea/ [4]	Confounding adjustment/cut-off details often NR; cohort/platform heterogeneity; limited independent prospective validation
TNFRSF10C	Tier 4	DFS	No (KM/association)	Internal (single clinical cohort)	NGS (WES)	Surveillance stratification; adjuvant consideration	NR	Yang X et al./2021/ China[2]	Confounding adjustment/cut-off details often NR; cohort/platform heterogeneity; limited independent prospective validation
hsa-miR-1229-3p	Tier 4	OS	No (KM/association)	Internal (single public cohort: TCGA)	NGS (RNA-seq)	Adjuvant intensity; surveillance stratification	NR	Li et al./2019/ China[18]	Confounding adjustment/cut-off details often NR; cohort/platform heterogeneity; limited independent prospective validation

Table S6. List of studies excluded at the full-text screening stage and reasons for exclusion.

	Title	Year	Journal	First author	Exclusion Reasons
1 [21]	lncKRT16P6 promotes tongue squamous cell carcinoma progression by sponging miR-3180 and regulating GATAD2A expression	2022	International journal of oncology	Zhang M.	non-omic design
2 [22]	Upregulated long non-coding RNA LINC00152 expression is associated with progression and poor prognosis of tongue squamous cell carcinoma.	2017	Journal of Cancer	Yu J.	Inadequate evaluation of prognosis
3 [23]	Functional analysis of lncRNAs based on competitive endogenous RNA in tongue squamous cell carcinoma.	2019	PeerJ	Song Y.	Inadequate evaluation of prognosis
4 [24]	Proteomic Analysis of Circulating Extracellular Vesicles Identifies Potential Biomarkers for Lymph Node Metastasis in Oral Tongue Squamous Cell Carcinoma.	2021	Cells	Qu X.	wrong population
5 [25]	Mutation-associated transcripts reconstruct the prognostic features of oral tongue squamous cell carcinoma.	2023	International journal of oral science	Liang L.	Lack of prognostic focus
6 [26]	Novel Prognostic Model Construction of Tongue Squamous Cell Carcinoma Based on Apigenin-Associated Genes.	2024	Frontiers in bioscience (Landmark edition)	Lai J.	non-omic design
7 [27]	A Minimal DNA Methylation Signature in Oral Tongue Squamous Cell Carcinoma Links Altered Methylation with Tumor Attributes.	2016	Molecular cancer research : MCR	Krishnan NM.	Inadequate evaluation of prognosis
8 [28]	Distinctive pattern of let-7 family microRNAs in aggressive carcinoma of the oral tongue in young patients.	2016	Oncology letters	Hilly O.	non-omic design
9 [29]	Increased expression of the long non-coding RNA UCA1 in tongue squamous cell carcinomas: a possible correlation with cancer metastasis.	2014	Oral surgery, oral medicine, oral pathology and oral radiology	Fang Z.	non-omic design
10 [30]	Role of miR-944/MMP10/AXL- axis in lymph node metastasis in tongue cancer.	2023	Communications biology	Dharavath B.	non-omic design
11 [31]	Identification of prognostic biomarkers for early detection of tongue squamous cell carcinoma: A systematic retrospective analysis	2023	Human Gene	Bhavsar M.	Inadequate evaluation of prognosis
12 [32]	A PROGNOSTIC FIVE-LNCRNA EXPRESSION SIGNATURE FOR SURVIVAL PREDICTION IN PATIENTS WITH TONGUE SQUAMOUS CELL CARCINOMA	2022	Acta Medica Mediterranea	Yu Z.	Full text could not be retrieved

13 [33]	Bioinformatic screening and experimental analysis identify SFRP1 as a prognostic biomarker for tongue squamous cell carcinomas	2020	Archives of Oral Biology	Yu M.	Inadequate evaluation of prognosis
14 [34]	Analysis of Differentially Expressed Long Non-coding RNAs and the Associated TF-mRNA Network in Tongue Squamous Cell Carcinoma	2020	Frontiers in Oncology	Zhang M.	wrong population
15 [35]	Decreased expression of mitochondrial mir-5787 contributes to chemoresistance by reprogramming glucose metabolism and inhibiting MT-CO3 translation	2019	Theranostics	Chen W.	wrong population
16 [36]	Global Quantitative Proteomics reveal Deregulation of Cytoskeletal and Apoptotic Signalling Proteins in Oral Tongue Squamous Cell Carcinoma	2018	Scientific Reports	Ananthi S.	Lack of prognostic focus
17 [37]	Characterizing Genetic Transitions of Copy Number Alterations and Allelic Imbalances in Oral Tongue Carcinoma Metastasis	2016	Genes Chromosomes and Cancer	Morita T.	Inadequate evaluation of prognosis
18 [38]	Long non-coding RNA deregulation in tongue squamous cell carcinoma	2014	BioMed Research International	Gao W.	Inadequate evaluation of prognosis
19 [39]	Integrated analysis of oral tongue squamous cell carcinoma identifies key variants and pathways linked to risk habits, HPV, clinical parameters and tumor recurrence	2015	F1000Research	Panda B.	Inadequate evaluation of prognosis
20 [40]	Tongue carcinoma infrequently harbor common actionable genetic alterations	2014	BMC Cancer	Tan D.S.W.	non-omic design
21 [41]	Comprehensive analysis of lncRNA-associated competing endogenous RNA network in tongue squamous cell carcinoma	2019	PeerJ	Zhang S.	Inadequate evaluation of prognosis
22 [42]	FSCN1 is an effective marker of poor prognosis and a potential therapeutic target in human tongue squamous cell carcinoma	2019	Cell Death & Disease	Chen Y.	Inadequate evaluation of prognosis
23 [43]	SEMA3A Exon 9 Expression Is a Potential Prognostic Marker of Unfavorable Recurrence-Free Survival in Patients with Tongue Squamous Cell Carcinoma	2020	DNA and Cell Biology	Tian T.	non-omic design
24 [44]	An Integrated Analysis of Prognostic Signature and Immune Microenvironment in Tongue Squamous Cell Carcinoma	2022	Frontiers in Oncology	Jin Y.	Lack of prognostic focus
25 [45]	Molecular characterisation in tongue squamous cell carcinoma reveals key variants potentially linked to clinical outcomes	2020	Cancer Biomarkers	Alsofyani A.	Lack of prognostic focus

26 [46]	Validation of Selected Head and Neck Cancer Prognostic Markers from the Pathology Atlas in an Oral Tongue Cancer Cohort	2021	Cancers	Wirsing A.	wrong site
27 [47]	Construction and validation of a prognostic model for tongue cancer based on three genes signature	2023	Medicine	Tan H.	wrong site
28 [48]	Four PTEN-targeting co-expressed miRNAs and ACTN4-targeting miR-548b are independent prognostic biomarkers in human squamous cell carcinoma of the oral tongue	2017	International Journal of Cancer	Berania I.	non-omic design
29 [49]	Genomic characterization of tobacco/nut chewing HPV-negative early stage tongue tumors identify MMP10 as a candidate to predict metastases	2017	Oral Oncology	Upadhyay P.	non-omic design
30 [50]	Signatures and prognostic values of related immune targets in tongue cancer	2023	Frontiers in Surgery	Lv X.	Inadequate evaluation of prognosis
31 [51]	Identification of ITGA3 as an Oncogene in Human Tongue Cancer via Integrated Bioinformatics Analysis	2018	Current Medical Science	Chen W.	Inadequate evaluation of prognosis

Table S7. PRISMA 2020 for Abstracts Checklist [52]

Section and Topic	Item #	Checklist item	Reported (Yes/No)
**TITLE**			
Title	1	Identify the report as a systematic review.	2-4
**BACKGROUND**			
Objectives	2	Provide an explicit statement of the main objective(s) or question(s) the review addresses.	21-25
**METHODS**			
Eligibility criteria	3	Specify the inclusion and exclusion criteria for the review.	21-28
Information sources	4	Specify the information sources (e.g. databases, registers) used to identify studies and the date when each was last searched.	26-27
Risk of bias	5	Specify the methods used to assess risk of bias in the included studies.	28-30
Synthesis of results	6	Specify the methods used to present and synthesise results.	28
**RESULTS**			
Included studies	7	Give the total number of included studies and participants and summarise relevant characteristics of studies.	30
Synthesis of results	8	Present results for main outcomes, preferably indicating the number of included studies and participants for each. If meta-analysis was done, report the summary estimate and confidence/credible interval. If comparing groups, indicate the direction of the effect (i.e. which group is favoured).	30-36
**DISCUSSION**			
Limitations of evidence	9	Provide a brief summary of the limitations of the evidence included in the review (e.g. study risk of bias, inconsistency and imprecision).	36-37
Interpretation	10	Provide a general interpretation of the results and important implications.	34-38
**OTHER**			
Funding	11	Specify the primary source of funding for the review.	974
Registration	12	Provide the register name and registration number.	982-983

Table S8. PRISMA 2020 Checklist [52]

Section and Topic	Item #	Checklist item	Location where item is reported
**TITLE**			
Title	1	Identify the report as a systematic review.	1-4
**ABSTRACT**			
Abstract	2	See the PRISMA 2020 for Abstracts checklist.	20-38
**INTRODUCTION**			
Rationale	3	Describe the rationale for the review in the context of existing knowledge.	59-78
Objectives	4	Provide an explicit statement of the objective(s) or question(s) the review addresses.	106-125
**METHODS**			
Eligibility criteria	5	Specify the inclusion and exclusion criteria for the review and how studies were grouped for the syntheses.	133-179
Information sources	6	Specify all databases, registers, websites, organisations, reference lists and other sources searched or consulted to identify studies. Specify the date when each source was last searched or consulted.	183-187
Search strategy	7	Present the full search strategies for all databases, registers and websites, including any filters and limits used.	183-195 & Fiqures 1, S1, S2, S3
Selection process	8	Specify the methods used to decide whether a study met the inclusion criteria of the review, including how many reviewers screened each record and each report retrieved, whether they worked independently, and if applicable, details of automation tools used in the process.	204-209
Data collection process	9	Specify the methods used to collect data from reports, including how many reviewers collected data from each report, whether they worked independently, any processes for obtaining or confirming data from study investigators, and if applicable, details of automation tools used in the process.	211-212 & Table S4
Data items	10a	List and define all outcomes for which data were sought. Specify whether all results that were compatible with each outcome domain in each study were sought (e.g. for all measures, time points, analyses), and if not, the methods used to decide which results to collect.	212-217
	10b	List and define all other variables for which data were sought (e.g. participant and intervention characteristics, funding sources). Describe any assumptions made about any missing or unclear information.	217-222
Study risk of bias assessment	11	Specify the methods used to assess risk of bias in the included studies, including details of the tool(s) used, how many reviewers assessed each study and whether they worked independently, and if applicable, details of automation tools used in the process.	224-240
Effect measures	12	Specify for each outcome the effect measure(s) (e.g. risk ratio, mean difference) used in the synthesis or presentation of results.	258-261
Synthesis methods	13a	Describe the processes used to decide which studies were eligible for each synthesis (e.g. tabulating the study intervention characteristics and comparing against the planned groups for each synthesis (item #5)).	242-257

Section and Topic	Item #	Checklist item	Location where item is reported
	13b	Describe any methods required to prepare the data for presentation or synthesis, such as handling of missing summary statistics, or data conversions.	270-272
	13c	Describe any methods used to tabulate or visually display results of individual studies and syntheses.	258-277
	13d	Describe any methods used to synthesize results and provide a rationale for the choice(s). If meta-analysis was performed, describe the model(s), method(s) to identify the presence and extent of statistical heterogeneity, and software package(s) used.	250-257
	13e	Describe any methods used to explore possible causes of heterogeneity among study results (e.g. subgroup analysis, meta-regression).	na
	13f	Describe any sensitivity analyses conducted to assess robustness of the synthesized results.	na
Reporting bias assessment	14	Describe any methods used to assess risk of bias due to missing results in a synthesis (arising from reporting biases).	Table S1, Table S2.
Certainty assessment	15	Describe any methods used to assess certainty (or confidence) in the body of evidence for an outcome.	269-272
**RESULTS**			
Study selection	16a	Describe the results of the search and selection process, from the number of records identified in the search to the number of studies included in the review, ideally using a flow diagram.	344-354 & Figure 2.
	16b	Cite studies that might appear to meet the inclusion criteria, but which were excluded, and explain why they were excluded.	Table S6
Study characteristics	17	Cite each included study and present its characteristics.	389-539 & Table 2.
Risk of bias in studies	18	Present assessments of risk of bias for each included study.	545-570 & Table S1, Table S2
Results of individual studies	19	For all outcomes, present, for each study: (a) summary statistics for each group (where appropriate) and (b) an effect estimate and its precision (e.g. confidence/credible interval), ideally using structured tables or plots.	Tables 3, 4, 5
Results of syntheses	20a	For each synthesis, briefly summarise the characteristics and risk of bias among contributing studies.	Tables 3, 4, 5
	20b	Present results of all statistical syntheses conducted. If meta-analysis was done, present for each the summary estimate and its precision (e.g. confidence/credible interval) and measures of statistical heterogeneity. If comparing groups, describe the direction of the effect.	na
	20c	Present results of all investigations of possible causes of heterogeneity among study results.	na
	20d	Present results of all sensitivity analyses conducted to assess the robustness of the synthesized results.	na

Section and Topic	Item #	Checklist item	Location where item is reported
Reporting biases	21	Present assessments of risk of bias due to missing results (arising from reporting biases) for each synthesis assessed.	Table S1, Table S2
Certainty of evidence	22	Present assessments of certainty (or confidence) in the body of evidence for each outcome assessed.	Table 3
**DISCUSSION**			
Discussion	23a	Provide a general interpretation of the results in the context of other evidence.	656-851
	23b	Discuss any limitations of the evidence included in the review.	899-911
	23c	Discuss any limitations of the review processes used.	942-948
	23d	Discuss implications of the results for practice, policy, and future research.	927-939
**OTHER INFORMATION**			
Registration and protocol	24a	Provide registration information for the review, including register name and registration number, or state that the review was not registered.	982-983
	24b	Indicate where the review protocol can be accessed, or state that a protocol was not prepared.	983
	24c	Describe and explain any amendments to information provided at registration or in the protocol.	na
Support	25	Describe sources of financial or non-financial support for the review, and the role of the funders or sponsors in the review.	974
Competing interests	26	Declare any competing interests of review authors.	981
Availability of data, code and other materials	27	Report which of the following are publicly available and where they can be found: template data collection forms; data extracted from included studies; data used for all analyses; analytic code; any other materials used in the review.	na

References

1. Hayden, J.A.; van der Windt, D.A.; Cartwright, J.L.; Côté, P.; Bombardier, C. Assessing Bias in Studies of Prognostic Factors. https://doi.org/10.7326/0003-4819-158-4-201302190-00009 2013, 158, 280–286, doi:10.7326/0003-4819-158-4-201302190-00009.
2. Yang, X.; Fang, L.; Zhang, C. CNV Analysis of the Correlation between Preoperative Lymph Node Metastasis and Prognosis of Early Tongue Cancer. J Cancer 2021, 12, 6135–6144, doi:10.7150/jca.60447.
3. Xiao, F.; Dai, Y.; Hu, Y.; Lu, M.; Dai, Q. Expression Profile Analysis Identifies IER3 to Predict Overall Survival and Promote Lymph Node Metastasis in Tongue Cancer. Cancer Cell Int 2019, 19, doi:10.1186/s12935-019-1028-2.

4. Lee, D.Y.; Hun Hah, J.; Jeong, W.J.; Chung, E.J.; Kwon, T.K.; Ahn, S.H.; Sung, M.W.; Kwon, S.K. The Expression of Defensin-Associated Genes May Be Correlated With Lymph Node Metastasis of Early-Stage Tongue Cancer. Clin Exp Otorhinolaryngol 2022, 15, 372–379, doi:10.21053/ceo.2022.00150.

5. Lee, D.Y.; Kang, Y.; Im, N.R.; Kim, B.; Kwon, T.K.; Jung, K.Y.; Baek, S.K. Actin-Associated Gene Expression Is Associated with Early Regional Metastasis of Tongue Cancer. Laryngoscope 2021, 131, 813–819, doi:10.1002/lary.29025.

6. Yang, X.; Wu, K.; Li, S.; Hu, L.; Han, J.; Zhu, D.; Tian, X.; Liu, W.; Tian, Z.; Zhong, L.; et al. MFAP5 and TNNC1: Potential Markers for Predicting Occult Cervical Lymphatic Metastasis and Prognosis in Early Stage Tongue Cancer; 2017; Vol. 8;.

7. Li, Y.; Wan, Q.; Wang, W.; Mai, L.; Sha, L.; Mashrah, M.; Lin, Z.; Pan, C. LncRNA ADAMTS9-AS2 Promotes Tongue Squamous Cell Carcinoma Proliferation, Migration and EMT via the MiR-600/EZH2 Axis. Biomedicine and Pharmacotherapy 2019, 112, doi:10.1016/j.biopha.2019.108719.

8. Kim, S.; Lee, C.; Kim, H.; Yoon, S.O. Genetic Characteristics of Advanced Oral Tongue Squamous Cell Carcinoma in Young Patients. Oral Oncol 2023, 144, doi:10.1016/j.oraloncology.2023.106466.

9. Liu, H.; Li, Q.; Qi, H.; Du, F.; Qiu, Y. Identification of Circular RNA_0000919 as a Potential Diagnostic and Prognostic Biomarker of Tongue Squamous Cell Carcinoma Using Circular RNA Microarray and Reverse Transcription-Quantitative PCR Analyses. Oncol Lett 2022, 24, doi:10.3892/ol.2022.13390.

10. Dou, H.; Song, C.; Wang, X.; Feng, Z.; Su, Y.; Wang, H. Integrated Bioinformatics Analysis of SEMA3C in Tongue Squamous Cell Carcinoma Using Machine-Learning Strategies. Cancer Cell Int 2024, 24, doi:10.1186/s12935-024-03247-y.

11. Thangaraj, S.V.; Shyamsundar, V.; Krishnamurthy, A.; Ramshankar, V. Deregulation of Extracellular Matrix Modeling with Molecular Prognostic Markers Revealed by Transcriptome Sequencing and Validations in Oral Tongue Squamous Cell Carcinoma. Sci Rep 2021, 11, doi:10.1038/s41598-020-78624-4.

12. Wang, S.; Fu, Z.; Wang, Y.; Sun, Y.; Cui, L.; Wang, C.; Liu, Q.; Shao, D.; Wang, Y.; Wen, N. Correlation of Carbonic Anhydrase 9 (CA9) with Pathological T-Stage and Prognosis in Patients with Oral Tongue Squamous Cell Carcinoma. *Ann Transl Med* 2020, *8*, 1521–1521, doi:10.21037/atm-20-7144.

13. Wolff, R.F.; Moons, K.G.M.; Riley, R.D.; Whiting, P.F.; Westwood, M.; Collins, G.S.; Reitsma, J.B.; Kleijnen, J.; Mallett, S. PROBAST: A Tool to Assess the Risk of Bias and Applicability of Prediction Model Studies. https://doi.org/10.7326/M18-1376 2019, 170, 51–58, doi:10.7326/M18-1376.

14. Ren, Y.; Da, J.; Ren, J.; Song, Y.; Han, J. An Autophagy-Related Long Non-Coding RNA Signature in Tongue Squamous Cell Carcinoma. BMC Oral Health 2023, 23, doi:10.1186/s12903-023-02806-5.

15. Liu, M.; Tong, L.; Liang, B.; Song, X.; Xie, L.; Peng, H.; Huang, D. A 15-Gene Signature and Prognostic Nomogram for Predicting Overall Survival in Non-Distant Metastatic Oral Tongue Squamous Cell Carcinoma. Front Oncol 2021, 11, doi:10.3389/fonc.2021.587548.

16. Zhu, H.; Tao, Y.; Huang, Q.; Chen, Z.; Jiang, L.; Yan, H.; Zhong, J.; Liang, L. Identification of Ferroptosis-Related Genes as Potential Biomarkers of Tongue Squamous Cell Carcinoma Using an Integrated Bioinformatics Approach. FEBS Open Bio 2022, 12, 412–429, doi:10.1002/2211-5463.13348.

17. Hu, D.; Messadi, D. V. Immune-Related Long Non-Coding RNA Signatures for Tongue Squamous Cell Carcinoma. Current Oncology 2023, 30, 4817–4832, doi:10.3390/curroncol30050363.

18. Li, Z.; Jiang, C.; Yuan, Y. TCGA Based Integrated Genomic Analyses of CeRNA Network and Novel Subtypes Revealing Potential Biomarkers for the Prognosis and Target Therapy of Tongue Squamous Cell Carcinoma. PLoS One 2019, 14, doi:10.1371/journal.pone.0216834.

19. Jin, Y.; Wang, Z.; He, D.; Zhu, Y.; Chen, X.; Cao, K. Identification of Novel Subtypes Based on SsGSEA in Immune-Related Prognostic Signature for Tongue Squamous Cell Carcinoma. Cancer Med 2021, 10, 8693–8707, doi:10.1002/cam4.4341.

20. McShane, L.M.; Altman, D.G.; Sauerbrei, W.; Taube, S.E.; Gion, M.; Clark, G.M.; Costa, J.; Di Leo, A.; Mayer, R.J. Reporting Recommendations for Tumor Marker Prognostic Studies (REMARK). J Natl Cancer Inst 2005, 97, 1180–1184, doi:10.1093/jnci/dji237.

21. Zhang M, Wu L, Wang X, Chen J. lncKRT16P6 promotes tongue squamous cell carcinoma progression by sponging miR-3180 and regulating GATAD2A expression. *Int J Oncol*. 2022;61(3):111. doi:10.3892/ijo.2022.5401

22. Yu J, Liu Y, Guo C, et al. Upregulated long non-coding RNA LINC00152 expression is associated with progression and poor prognosis of tongue squamous cell carcinoma. *J Cancer*. 2017;8(4):523-530. Published 2017 Feb 11. doi:10.7150/jca.17510

23. Song Y, Pan Y, Liu J. Functional analysis of lncRNAs based on competitive endogenous RNA in tongue squamous cell carcinoma. *PeerJ*. 2019;7:e6991. Published 2019 May 28. doi:10.7717/peerj.6991

24. Qu X, Leung TCN, Ngai SM, et al. Proteomic Analysis of Circulating Extracellular Vesicles Identifies Potential Biomarkers for Lymph Node Metastasis in Oral Tongue Squamous Cell Carcinoma. *Cells*. 2021;10(9):2179. Published 2021 Aug 24. doi:10.3390/cells10092179

25. Liang L, Li Y, Ying B, et al. Mutation-associated transcripts reconstruct the prognostic features of oral tongue squamous cell carcinoma. *Int J Oral Sci*. 2023;15(1):1. Published 2023 Jan 3. doi:10.1038/s41368-022-00210-3

26. Lai J, Fang C, Zhang G, et al. Novel Prognostic Model Construction of Tongue Squamous Cell Carcinoma Based on Apigenin-Associated Genes. *Front Biosci (Landmark Ed)*. 2024;29(2):65. doi:10.31083/j.fbl2902065

27. Krishnan NM, Dhas K, Nair J, et al. A Minimal DNA Methylation Signature in Oral Tongue Squamous Cell Carcinoma Links Altered Methylation with Tumor Attributes. *Mol Cancer Res*. 2016;14(9):805-819. doi:10.1158/1541-7786.MCR-15-0395

28. Hilly O, Pillar N, Stern S, et al. Distinctive pattern of let-7 family microRNAs in aggressive carcinoma of the oral tongue in young patients. *Oncol Lett*. 2016;12(3):1729-1736. doi:10.3892/ol.2016.4892

29. Fang Z, Wu L, Wang L, Yang Y, Meng Y, Yang H. Increased expression of the long non-coding RNA UCA1 in tongue squamous cell carcinomas: a possible correlation with cancer metastasis. *Oral Surg Oral Med Oral Pathol Oral Radiol*. 2014;117(1):89-95. doi:10.1016/j.oooo.2013.09.007

30. Dharavath B, Butle A, Pal A, et al. Role of miR-944/MMP10/AXL- axis in lymph node metastasis in tongue cancer. *Commun Biol*. 2023;6(1):57. Published 2023 Jan 17. doi:10.1038/s42003-023-04437-6

31. Bhavsar, M.; Patel, S.; Rawal, R.; Patel, S.; Mankad, A. Identification of Prognostic Biomarkers for Early Detection of Tongue Squamous Cell Carcinoma: A Systematic Retrospective Analysis. Human Gene 2023, 38, doi:10.1016/j.humgen.2023.201234.

32. Yu, M.; Wu, G.; Chen, Y.; Wang, H.; Gao, Y.; Wang, A. Bioinformatic Screening and Experimental Analysis Identify SFRP1 as a Prognostic Biomarker for Tongue Squamous Cell Carcinomas. Arch. Oral Biol. 2020, 110, doi:10.1016/j.archoralbio.2019.104587.

33. Yu, Z.; Xia, L.; Leng, W.; Shi, J.; He, W.; Mao, M.; Chai, H.; Yan, Y. A Prognostic five lnc-RNA Expression Signature for Survival Prediction in Patients with Tongue Squamous Cell Carcinoma. Acta Medica Mediterranea 2022, 38, 1641–1648, doi:10.19193/0393-6384_2022_3_249.

34. Zhang, M.; Chen, Z.; Zhang, S.; Wu, L.; Jie, Y.; Liao, Y.; Huang, Y.; Chen, J.; Shi, B. Analysis of Differentially Expressed Long Non-Coding RNAs and the Associated TF-MRNA Network in Tongue Squamous Cell Carcinoma. Front. Oncol. 2020, 10, doi:10.3389/fonc.2020.01421.

35. Chen, W.; Wang, P.; Lu, Y.; Jin, T.; Lei, X.; Liu, M.; Zhuang, P.; Liao, J.; Lin, Z.; Li, B.; et al. Decreased Expression of Mitochondrial Mir-5787 Contributes to Chemoresistance by Reprogramming Glucose Metabolism and Inhibiting MT-CO3 Translation. Theranostics 2019, 9, 5739–5754, doi:10.7150/thno.37556.

36. Ananthi, S.; Lakshmi, C.N.P.; Atmika, P.; Anbarasu, K.; Mahalingam, S. Global Quantitative Proteomics Reveal Deregulation of Cytoskeletal and Apoptotic Signalling Proteins in Oral Tongue Squamous Cell Carcinoma. Sci. Rep. 2018, 8, doi:10.1038/s41598-018-19937-3.

37. Morita, T.; Uzawa, N.; Mogushi, K.; Sumino, J.; Michikawa, C.; Takahashi, K.I.; Myo, K.; Izumo, T.; Harada, K. Characterizing Genetic Transitions of Copy Number Alterations and Allelic Imbalances in Oral Tongue Carcinoma Metastasis. Genes Chromosomes Cancer 2016, 55, 975–986, doi:10.1002/gcc.22395.

38. Gao, W.; Chan, J.Y.W.; Wong, T.S. Long Non-Coding RNA Deregulation in Tongue Squamous Cell Carcinoma. Biomed Res. Int. 2014, 2014, doi:10.1155/2014/405860.

39. Panda, B.; Krishnan, N.; Gupta, S.; Palve, V.; Varghese, L.; Pattnaik, S.; Jain, P.; Khyriem, C.; Hariharan, A.; Dhas, K.; et al. Integrated Analysis of Oral Tongue Squamous Cell Carcinoma Identifies Key Variants and Pathways Linked to Risk Habits, HPV, Clinical Parameters and Tumor Recurrence. F1000Res. 2015, 4, doi:10.12688/f1000research.7302.1.

40. Tan, D.S.W.; Wang, W.; Leong, H.S.; Sew, P.H.; Lau, D.P.; Chong, F.T.; Krisna, S.S.; Lim, T.K.H.; Iyer, N.G. Tongue Carcinoma Infrequently Harbor Common Actionable Genetic Alterations. BMC Cancer 2014, 14, doi:10.1186/1471-2407-14-679.

41. Zhang, S.; Cao, R.; Li, Q.; Yao, M.; Chen, Y.; Zhou, H. Comprehensive Analysis of LncRNA-Associated Competing Endogenous RNA Network in Tongue Squamous Cell Carcinoma. PeerJ 2019, 2019, doi:10.7717/peerj.6397.

42. Chen, Y.; Tian, T.; Li, Z.Y.; Wang, C.Y.; Deng, R.; Deng, W.Y.; Yang, A. kui; Chen, Y.F.; Li, H. FSCN1 Is an Effective Marker of Poor Prognosis and a Potential Therapeutic Target in Human Tongue Squamous Cell Carcinoma. Cell Death Dis. 2019, 10, doi:10.1038/s41419-019-1574-5.

43. Tian, T.; Zhang, L.; Tang, K.; Wang, A.; Wang, J.; Wang, J.; Wang, F.; Wang, W.; Ma, X. SEMA3A Exon 9 Expression Is a Potential Prognostic Marker of Unfavorable Recurrence-Free Survival in Patients with Tongue Squamous Cell Carcinoma. DNA Cell Biol. 2020, 39, 555–562, doi:10.1089/dna.2019.5109.

44. Jin, Y.; Wang, Z.; Tang, W.; Liao, M.; Wu, X.; Wang, H. An Integrated Analysis of Prognostic Signature and Immune Microenvironment in Tongue Squamous Cell Carcinoma. Front. Oncol. 2022, 12, doi:10.3389/fonc.2022.891716.

45. Alsofyani, A.A.; Dallol, A.; Farraj, S.A.; Alsiary, R.A.; Samkari, A.; Alhaj-Hussain, B.T.; Khan, J.A.; Al-Maghrabi, J.; Al-Khayyat, S.S.; Alkhatabi, H.; et al. Molecular Characterisation in Tongue Squamous Cell Carcinoma Reveals Key Variants Potentially Linked to Clinical Outcomes. Cancer Biomarkers 2020, 28, 213–220, doi:10.3233/CBM-190897.

46. Wirsing, A.M.; Bjerkli, I.H.; Steigen, S.E.; Rikardsen, O.; Magnussen, S.N.; Hegge, B.; Seppola, M.; Uhlin-Hansen, L.; Hadler-Olsen, E. Validation of Selected Head and Neck Cancer Prognostic Markers from the Pathology Atlas in an Oral Tongue Cancer Cohort. Cancers (Basel). 2021, 13, doi:10.3390/cancers13102387.

47. Tan, H.; Huang, H.; Yang, H.; Qian, J.; Wei, L.; Liu, W. Construction and Validation of a Prognostic Model for Tongue Cancer Based on Three Genes Signature. Medicine (United States) 2023, 102, E36097, doi:10.1097/MD.0000000000036097.

48. Berania, I.; Cardin, G.B.; Clément, I.; Guertin, L.; Ayad, T.; Bissada, E.; Nguyen-Tan, P.F.; Filion, E.; Guilmette, J.; Gologan, O.; et al. Four PTEN-Targeting Co-Expressed MiRNAs and ACTN4- Targeting MiR-548b Are Independent Prognostic Biomarkers in Human Squamous Cell Carcinoma of the Oral Tongue. Int. J. Cancer 2017, 141, 2318–2328, doi:10.1002/ijc.30915.

49. Upadhyay, P.; Gardi, N.; Desai, S.; Chandrani, P.; Joshi, A.; Dharavath, B.; Arora, P.; Bal, M.; Nair, S.; Dutt, A. Genomic Characterization of Tobacco/Nut Chewing HPV-Negative Early Stage Tongue Tumors Identify MMP10 as a Candidate to Predict Metastases. Oral Oncol. 2017, 73, 56–64, doi:10.1016/j.oraloncology.2017.08.003.

50. Lv, X.; Yu, X. Signatures and Prognostic Values of Related Immune Targets in Tongue Cancer. Front. Surg. 2023, 9, doi:10.3389/fsurg.2022.952389.

51. Chen, W. li; Wang, X. kang; Wu, W. Identification of ITGA3 as an Oncogene in Human Tongue Cancer via Integrated Bioinformatics Analysis. Curr. Med. Sci. 2018, 38, 714–720, doi:10.1007/s11596-018-1935-9.

52. Page, M.J.; McKenzie, J.E.; Bossuyt, P.M.; Boutron, I.; Hoffmann, T.C.; Mulrow, C.D.; Shamseer, L.; Tetzlaff, J.M.; Akl, E.A.; Brennan, S.E.; et al. The PRISMA 2020 Statement: An Updated Guideline for Reporting Systematic Reviews. BMJ 2021, 372, doi:10.1136/BMJ.N71..
